# TFE3, a potential therapeutic target for Spinal Cord Injury via augmenting autophagy flux and alleviating ER stress

**DOI:** 10.7150/thno.46566

**Published:** 2020-07-23

**Authors:** Kailiang Zhou, Zhilong Zheng, Yao Li, Wen Han, Jing Zhang, Yuqin Mao, Huanwen Chen, Wanying Zhang, Mi Liu, Ling Xie, Hongyu Zhang, Huazi Xu, Jian Xiao

**Affiliations:** 1 Department of Orthopaedics, The Second Affiliated Hospital and Yuying Children's Hospital of Wenzhou Medical University, Wenzhou 325000, China.; 2 Molecular Pharmacology Research Center, School of Pharmaceutical Science, Wenzhou Medical University, Wenzhou, 325000, China.; 3 University of Maryland School of Medicine, Baltimore, MD 21201, USA.

**Keywords:** TFE3, Autophagy, ER stress-induced apoptosis, AMPK signaling pathways, Spinal cord injury

## Abstract

**Background and Aim:** Increasing evidence suggests that spinal cord injury (SCI)-induced defects in autophagic flux may contribute to an impaired ability for neurological repair following injury. Transcription factor E3 (TFE3) plays a crucial role in oxidative metabolism, lysosomal homeostasis, and autophagy induction. Here, we investigated the role of TFE3 in modulating autophagy following SCI and explored its impact on neurological recovery.

**Methods:** Histological analysis via HE, Nissl and Mason staining, survival rate analysis, and behavioral testing via BMS and footprint analysis were used to determine functional recovery after SCI. Quantitative real-time polymerase chain reaction, Western blotting, immunofluorescence, TUNEL staining, enzyme-linked immunosorbent assays, and immunoprecipitation were applied to examine levels of autophagy flux, ER-stress-induced apoptosis, oxidative stress, and AMPK related signaling pathways. *In vitro* studies using PC12 cells were performed to discern the relationship between ROS accumulation and autophagy flux blockade.

**Results:** Our results showed that in SCI, defects in autophagy flux contributes to ER stress, leading to neuronal death. Furthermore, SCI enhances the production of reactive oxygen species (ROS) that induce lysosomal dysfunction to impair autophagy flux. We also showed that TFE3 levels are inversely correlated with ROS levels, and increased TFE3 levels can lead to improved outcomes. Finally, we showed that activation of TFE3 after SCI is partly regulated by AMPK-mTOR and AMPK-SKP2-CARM1 signaling pathways.

**Conclusions:** TFE3 is an important regulator in ROS-mediated autophagy dysfunction following SCI, and TFE3 may serve as a promising target for developing treatments for SCI.

## Introduction

Spinal cord injury (SCI) is one of the leading causes of long-term disability and death worldwide 1. In the United States alone, SCI has an annual incidence of 12,000-20,000 cases, affecting a total of nearly half a million patients 2. During SCI, neuronal cell death is an important pathological event contributing to neurological deficits 3. Mechanistically, the majority of neuronal cell death is not due to direct mechanical damage, but rather to damage-induced biochemical disruptions such as protein misfolding and oxidative stress as part of secondary injury 4, 5. Protein misfolding often triggers the endoplasmic reticulum (ER) stress response/unfolded protein response (UPR) in the ER as an attempt to maintain homeostasis 6. However, excessive ER stress induces apoptosis (labeled with the molecular hallmarks of CHOP and Caspase12) in neurons and oligodendrocytes, which significantly limits neural regeneration and repair after SCI 7, 8, 9. Despite playing a clear pathological role in SCI, the mechanism by which ER stress induces and promotes apoptosis during secondary injury remains unknown.

Autophagy is an intracellular lysosome-dependent pathway for degrading waste such as cytoplasmic proteins, protein aggregates, and damaged organelles 10. Autophagy is initiated by the formation of autophagosomes engulfing waste proteins and damaged organelles, followed by fusion with lysosomes where lysosomal proteases degrade the cargo material 11. Thus, autophagy plays an important role in the homeostasis of various cells and is a particularly critical process in terminally differentiated cells such as neurons 12, 13 It was reported that autophagy can be induced by ER stress via several canonical UPR pathways 14, and it is believed to have been evolved as a mechanism to dispose of misfolded proteins that cannot be degraded by ER-associated proteasomal degradation 15. Interestingly, accumulating evidence has also suggested that disrupted autophagy flux, the dynamic process of autophagy, encompassing the entire process of autophagy holistically 16, may potentiate ER stress and induce apoptosis in neurons after SCI 2, 3. Thus, bi-directional interactions between ER stress and autophagy likely play significant roles in the pathophysiology of SCI.

Mechanistically, studies have demonstrated that impairments of lysosomal function contribute to the blockade of autophagy flux in SCI 17, and reactive oxygen species, or ROS 2, such as O_2_^-^, H_2_O_2_, HO_2_·, ·OH, and their lipid peroxidation products may be potential culprits 18. ROS oxidize unsaturated fatty acids in lysosome membranes to permeate the membranes which further induce the extravasation of proteolytic enzymes leading to the disruption of lysosomal functions 19, 20. Extensive studies have demonstrated that ROS and the oxidative stress they induce play a critical role during the secondary injury of SCI 21, where pathological changes such as edema, hemorrhage, and hypoxia induce mitochondrial dysfunction and increase the activities of ROS-producing enzymes (likes xanthine-xanthine oxidase), resulting in vast ROS production 22. With the scavenging capacity of the antioxidant system exhausted, excessive ROS is accumulated in the spinal cord lesion, which likely results in lysosomal dysfunction and subsequent blockade of autophagy flux. Despite ample knowledge of ROS and autophagic pathways, their roles in the pathophysiology of SCI are less defined.

Many studies have reported that microphthalmia family of bHLH-LZ transcription factors (MiT/TFE), including MITF, TFEB, TFE3, and TFEC 23, are important for the maintenance of cellular physiological and pathological processes 24, 25. Among these transcription factors' many facets of biological functions, their roles in oxidative metabolism, lysosomal homeostasis, and autophagy induction have attracted the most attention in the SCI research. Several studies have provided evidence that during cellular stress, MITF, TFEB, TFE3, and TFEC all promote autophagosome-lysosome activity by binding to the CLEAR (Coordinated Lysosomal Expression and Regulation) element on a variety of autophagy and lysosomal genes such as* Atg5*, *Vps34*, *Lc3*, *Ctsd,* and* Lamp2* to induce their expression 26. Moreover, MITF, TFEB, TFE3, and TFEC can reduce intracellular ROS by increasing the abundance of antioxidant genes such as *Pgc1α*, *Gpx,* and *Sod* 23, 27. Therefore, these transcription factors may underly SCI pathophysiology and thus may be leveraged for therapeutic benefit. Our results revealed that TFE3 transcriptional level and its protein expressions peaked at day1 after SCI, a time point with grievous ROS accumulation and lysosomal dysfuction. Hence, we postulate that targeting TFE3 may both promote antophagy-lysosomal activities and inhibit ROS accumulation, to alleviate autophagy flux disruption in neurons after SCI. Current studies have shown that, under a variety of stress conditions, AMPK-mTOR and AMPK-SKP2-CARM1 signaling pathways are activated to induce TFEB into the nucleus to exert bio-activities 28, 29, 30. So it is necessary to verify whether TFE3 also be regulated by the signaling pathways after SCI.

In the current study, we postulate that in SCI, excessive ROS generation during secondary injury contributes to lysosomal dysfunction and subsequently leads to autophagy flux blockade and ER stress-induced apoptosis. Furthermore, we hypothesize that SCI causes changes in MiT/TFE transcriptional factors. Using pharmacological inhibitors, short hairpin RNA, *in vitro* models, and transgenic mice, we demonstrate that in SCI, excess accumulation of ROS resulted in lysosomal malfunction leading to blockage of autophagy flux and subsequent ER-stressed induced apoptosis of neurons. Furthermore, we show that in SCI, activation of TFE3 via the AMPK-mTOR and AMPK-SKP2-CARM1 signaling pathways mitigated autophagy flux disruption and prevented ER stress-induced apoptosis, leading to improved functional recovery after injury. Thus, our results suggest that TFE3 may serve as a potential therapeutic target for SCI.

## Results

### SCI induces autophagy flux blockade and ER stress-mediated apoptosis in neurons

**Figure 1:**
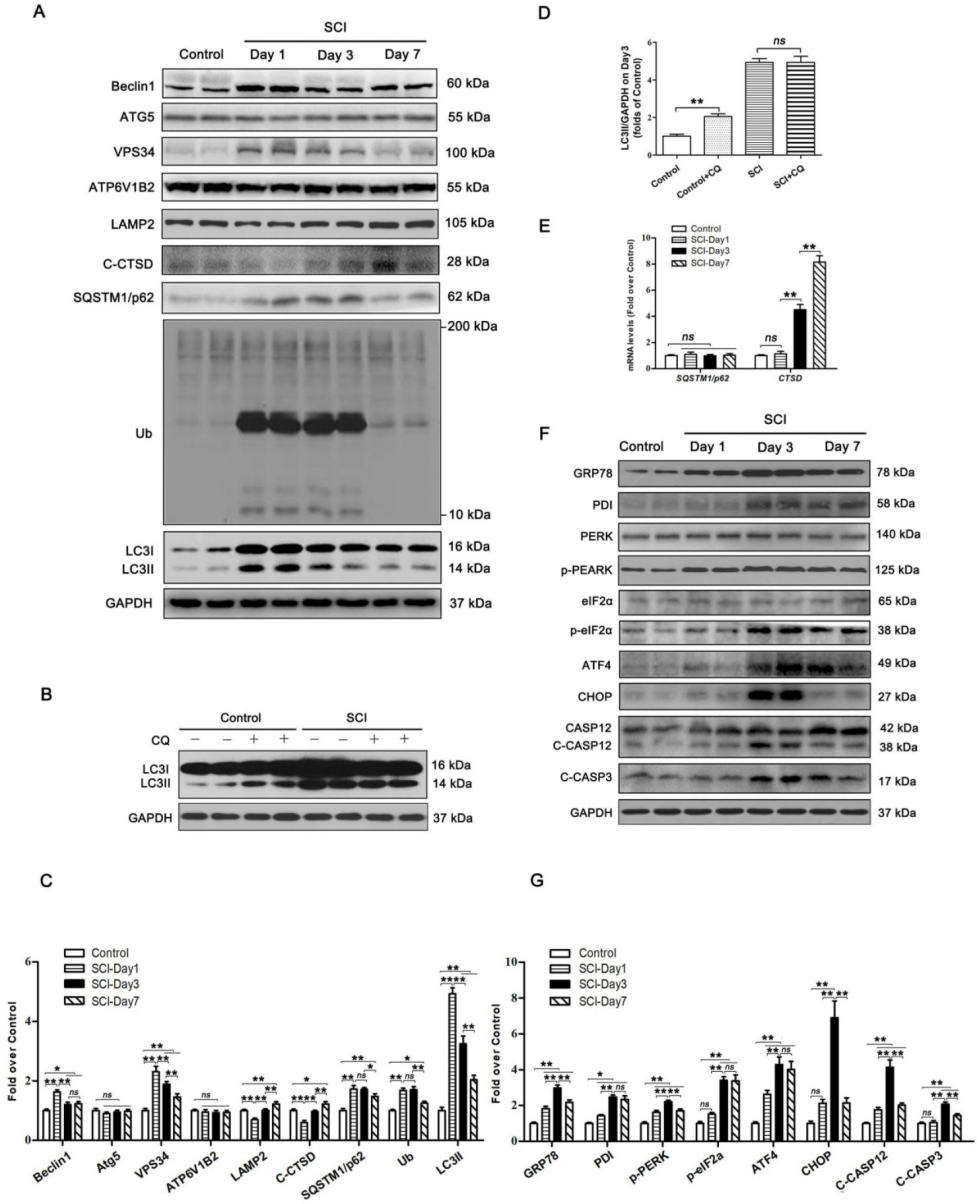
SCI leads to autophagy flux blockade and ER stress-induced apoptosis. (**A**) Western blotting of autophagy flux markers, Beclin1, ATG5, VPS34, ATP6V1B2, LAMP2, C-CTSD, SQSTM1/p62, UB and LC3 in spinal cord tissue from Control and SCI mice at the indicated time points. (**B**) Representative Western blotting of LC3 in Control and SCI (Day1) spinal cord slides cultured in the presence or absence of CQ. (**C**) Densitometric analysis of Beclin1, ATG5, VPS34, ATP6V1B2, LAMP2, C-CTSD, SQSTM1/p62, UB and LC3II data from (A) normalized to loading control GAPDH. (**D**) Densitometric analysis of LC3II from (C) normalized to the loading control GAPDH. (**E**) Relative mRNA level of *Sqstm1/p62* and *Ctsd* in the spinal cord from Control an SCI mice normalized to control *β-actin* at the indicated time points. (**F**) Western blot analysis of ER stress-induced apoptosis markers, GRP78, PDI, PERK, p-PERK, eIF2α, p-eIF2α, ATF4, CHOP, CASP12, C-CASP12, CASP3 and C-CASP3 in Control and SCI groups. (**G**) Densitometric analysis of GRP78, PDI, p-PERK, p-eIF2α, ATF4, CHOP, C-CASP12 and C-CASP3 from (F) normalized to loading control GAPDH. n=6, ns stands for not significant, *P<0.05, **P<0.01.

To examine the autophagy activity in neurons after SCI, we detected the protein levels of autophagosomal proteins (Beclin1, ATG5, VPS34, and LC3Ⅱ), lysosomal markers (ATP6V1B2, LAMP2, and CTSD), and autophagy substrate proteins (UB and SQSTM1/p62). As shown in Figure 1A and C, Becin1, VPS34, and LC3Ⅱ peaked at 1 day after SCI, and then gradually decreased by day 7. We also noticed a significant increase in the levels of UB and SQSTM1/p62 on days 1 and 3 followed by a decline on day 7. Immunofluorescence showed that LC3Ⅱ signals and p62 density in neurons peaked at day 1 and day 3 after injury, respectively; these levels then gradually decreased, but remained high at day 7 (Figure S1A-C). Analysis with qPCR demonstrated that *p62* mRNA levels did not increase on days 1, 3, and 7 following injury (Figure 1E). Together, these results indicate that autophagy flux was blocked, along with the activity of autophagy initiation, in neurons at 1 day after SCI. To further confirm this, we performed LC3 turnover assays of spinal cord slides. Results showed that chloroquine (CQ) treatment significantly increased LC3Ⅱ levels in the control group. However, the addition of CQ did not elevate LC3Ⅱ levels in the SCI-day 1 group (Figure 1B and D). Lysosomal function was examined as well. We found that CTSD and LAMP2 markedly decreased at day 1 following injury, returned to baseline levels by day 3, and significantly increased by day 7 (Figure 1A and C). A similar expression pattern for LAMP2 was observed in neurons following SCI (Figure S1A and D). However, *Ctsd* mRNA level was slightly increased at day 1 after injury, then further elevated at day 3 and 7 (Figure 1E). These data revealed that SCI results in impairment of lysosomal function, but not lysosomal biogenesis.

PERK, ATF6, and IRE-1, all three arms of the ER stress response signaling pathway, are all known to be activated after SCI 31. It also has been found that ablation of ATF6 does not affect locomotor recovery after spinal cord injury; and attenuation of PERK is a viable therapeutic target to improve functional recovery after SCI 32, 33. The function of IRE-1 inhibition in spinal cord injury has not been determined. These findings suggest that the PERK branch plays a crucial role in UPR after SCI. Therefore, we analyzed the PERK-CHOP signaling after SCI in the present study. Related biomarkers GRP78, PDI, PERK, p-PERK, eIF2α, p-eIF2α, ATF4, CHOP, CASP12, and C-CASP3 were measured. As shown in Figure 1F and G, the levels of GRP78, PDI, p-PERK, and C-CASP12 were elevated at day 1, peaked at day 3, and then decreased but remained substantially higher at day 7; expressions of eIF2α and ATF4 was slightly up-regulated at day 1, then further increased at day 3 and 7; CHOP and C-CASP3 expressions were not significantly increased at day 1 after injury, was notably elevated at day 3, and returned to the basal level at day 7. Immunofluorescence showed similar expression patterns: intensities of CHOP, CASP12 and CASP3 in neurons peaked at day 3 after injury, and gradually decreased by day 7 (Figure S1E-H). TUNEL staining demonstrated that the numbers of TUNEL-positive neurons starting to rise at day 1, peaked at day 3, and then decreased by day 7 after injury (Figure S1I-J). This revealed that ER stress-induced apoptosis was activated in neurons after SCI, with peak activity at day 3. Since ER stress-induced apoptosis peaked after autophagy flux blockade (day 3 vs. day 1, respectively), our data suggest that there may potentially be a causal relationship between disruption of autophagy and ER stress-induced apoptosis in neurons following SCI.

### Disruption of autophagy flux contributes to ER stress-induced apoptosis in neurons after SCI

**Figure 2:**
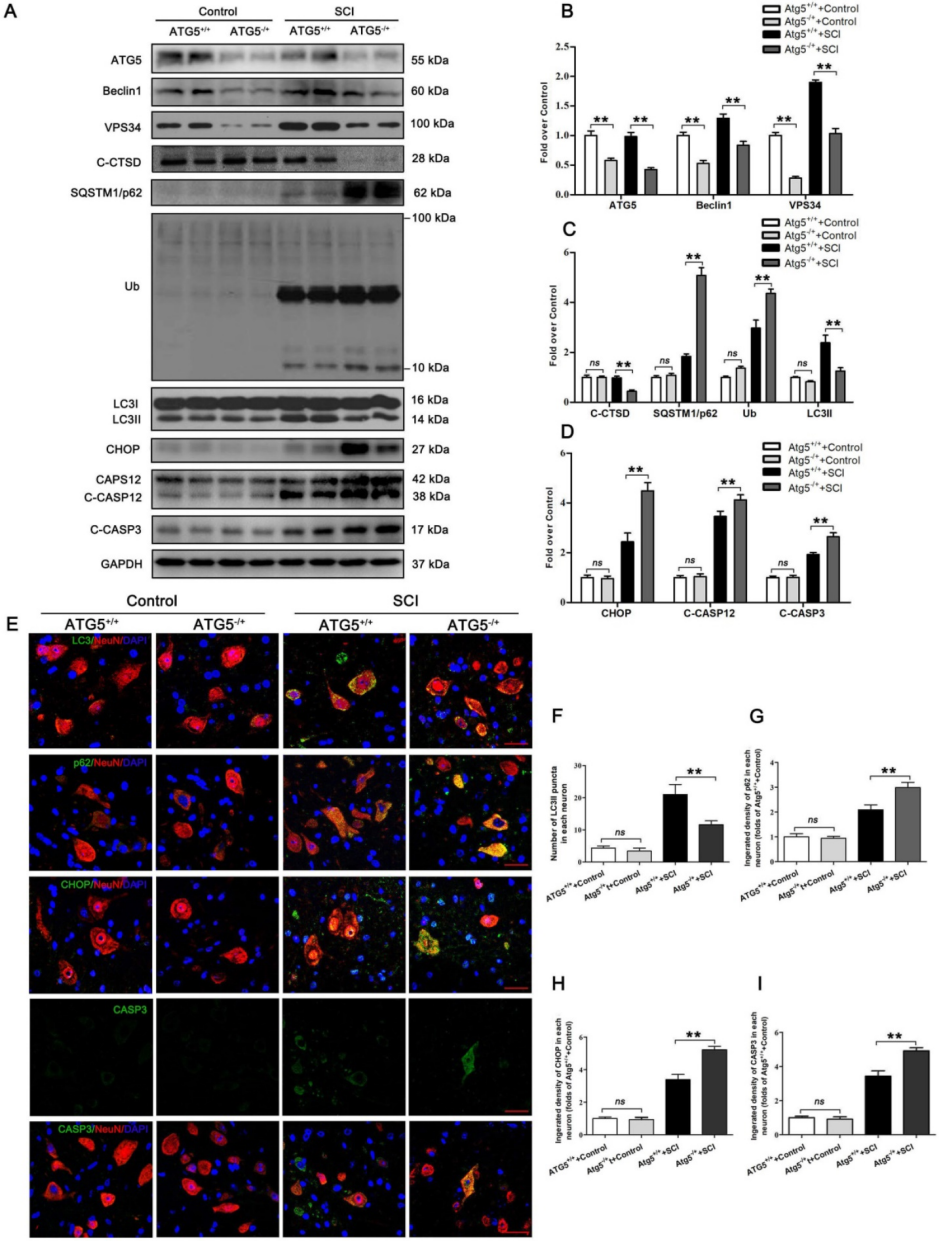
Inhibition of autophagy activity aggravates ER stress-induced apoptosis after SCI. (**A**) Western blot analysis of autophagy flux markers (ATG5, Beclin1, VPS34, C-CTSD, SQSTM1/p62, UB and LC3) and ER stress-induced apoptosis markers (CHOP, CASP12, C-CASP12, C-CASP3) in spinal cord lesions from ATG5-/+ mice and ATG5+/+ mice with and without SCI, at Day3 after SCI. (**B, C**) The levels of the autophagy flux makers from (A) normalized to loading control GAPDH. (**D**) The expressions of ER stress-induced apoptosis markers from (A) normalized to loading control GAPDH. (**E**) Image (30×) of spinal cord sections from the indicated groups at Day 3 stained with antibodies against LC3II/NeuN, p62/NeuN, CHOP/NeuN,and CASP3/NeuN, respectively. Scale bar: 25 µm. (**F**) Quantification of immunofluorescence data from (E) showing the mean number of LC3II in motor neurons at the spinal cord. (**G-I**) Quantification of immunofluorescence data from (E) showing the mean optical density of p62, CHOP, and CASP3, respectively, in motor neurons of spinal cord. n=6, ns stands for not significant, *P<0.05, **P<0.01.

We next used an ATG5 hemi-zygote knockout (ATG5^-/+^) mouse model to investigate whether disruption of autophagy flux would exacerbate ER stress-induced neuronal apoptosis after SCI. Here, we found that ATG5^-/+^ mice showed a decrease in autophagy flux compared to wild-type (ATG5^+/+^) mice after SCI at day 3. Consistently, expressions of ATG5, Beclin1, VPS34, C-CTSD, and LC3Ⅱ were all reduced. In addition, SQSTMQ/p62 and UB levels were persistently higher in ATG5^-/+^ mice (Figure 2A-C). Decreased flux was also corroborated by immunofluorescence showing less LC3Ⅱ signals number and more p62 density co-localization with neurons in ATG5^-/+^ mice on day 3 after SCI (Figure 2E-G). Together, these data suggest that impaired autophagy flux in neurons was further aggravated in ATG5^-/+^ mice after SCI.

CHOP, C-CASP12, CASP3, and C-CASP3 expression in neurons were notably elevated in ATG5^-/+^ mice on day 3 after SCI when compared with ATG5^+/+^ mice (Figure 2E, H and I). Compared to the ATG5^+/+^ mice, ATG5^-/+^ mice showed expanding area of glial scar (Figure S2A-B), less number of ventral motor neurons (Figure S2C-D), down-regulation MAP2 (Figure S2E-F), and decreasing number of SYN positive synapse onto ventral motor neurons following SCI (Figure S2G-H). The survival rate of ATG5^-/+^ mice was notably lower than ATG5+/+ mice (Figure S2I). Furthermore, BMS scores were also significantly decreased in ATG5^-/+^ mice after SCI at days 1, 3, 7, 14, 21, and 28, respectively (Figure S2J). At day 28 after injury, ATG5^+/+^ mice presented a significant restoration of hind legs movement with coordinated crawling, but ATG5^-/+^ mice were still dragging their hind legs (Figure S2K). These sets of experiments suggest that autophagy flux disruption contributes to ER stress-induced neuronal apoptosis and deterioration in functional recovery after SCI.

### ROS-induced lysosomal malfunction initiates autophagy flux disruption and ER stress-induced apoptosis in neurons after SCI

**Figure 3:**
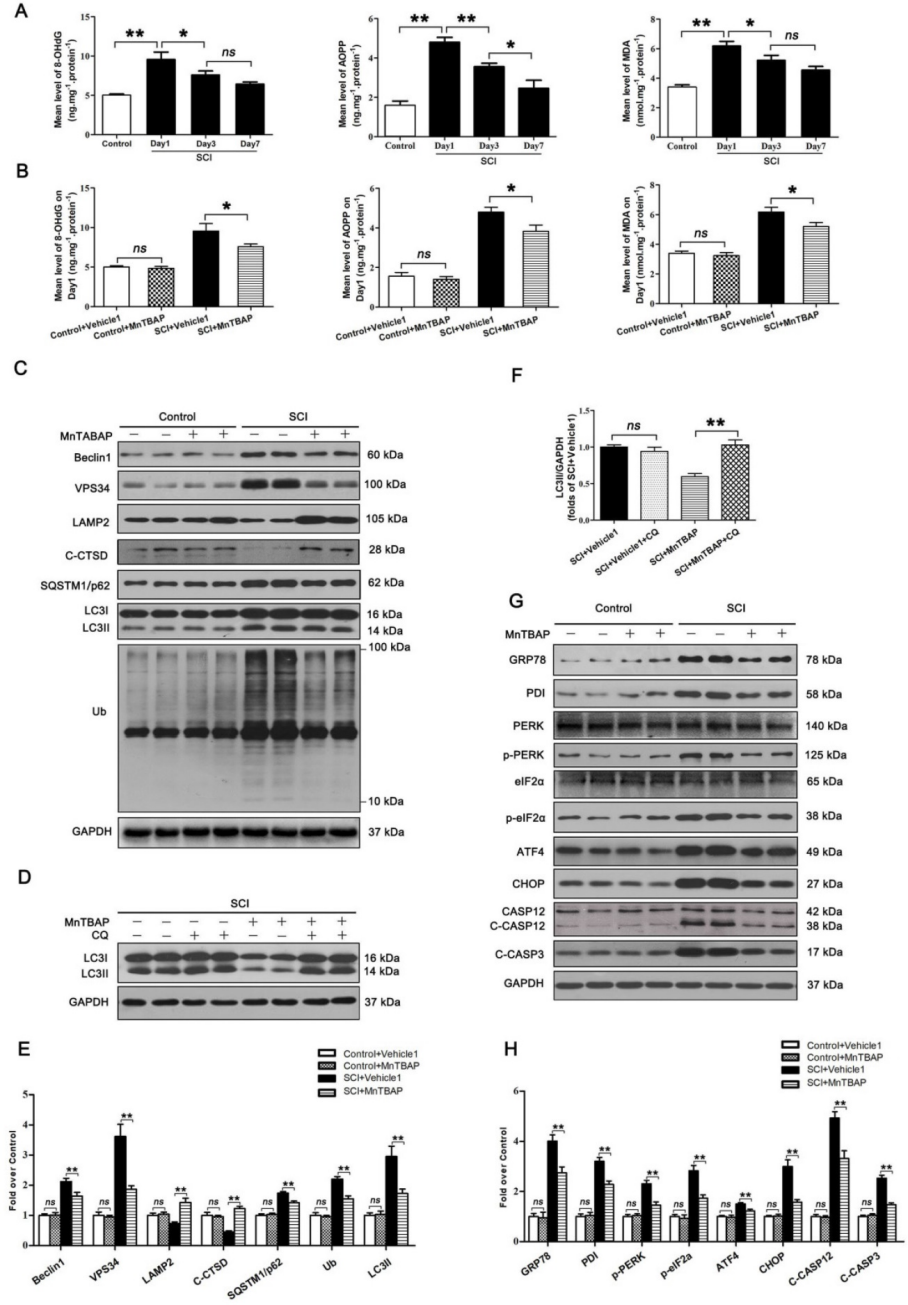
ROS-induced lysosomal dysfuntion initiates autophagy flux blockade and ER stress-induced apoptosis after SCI. (**A**) ELISA of oxidation products 8-OHdG, AOPP, and MDA in spinal cord lesions from Control and SCI mice at the indicated time points. (**B**) ELISA of 8-OHdG, AOPP, and MDA in spinal cord lesions from mice grouped as indicated at Day1 after SCI. (**C**) Western blotting of Beclin1, VPS34, C-CTSD, SQSTM1/p62, UB and LC3 in spinal cord from non-SCI (Control) mice and SCI mice treated with MnTBAP or Vehicle1 at Day1 after SCI. (**D**) Western blot analysis of LC3 in SCI+Vehicle1, and SCI treated with MnTBAP spinal cord slides at Day1 cultured in the presence or absence of CQ. (**E**) Densitometric analysis of Beclin1, VPS34, ATP6V1B2, LAMP2, C-CTSD, SQSTM1/p62, UB and LC3II from (C) normalized to loading control GAPDH. (**F**) Densitometric analysis of LC3II from (D) normalized to the loading control GAPDH. (**G**) Western blot analysis of GRP78, PDI, PERK, p-PERK, eIF2α, p-eIF2α, ATF4, CHOP, CASP12, C-CASP12, and C-CASP3 in spinal cord lesions from the grouped mice on Day3. (**H**) Densitometric analysis of GRP78, PDI, p-PERK, p-eIF2α, ATF4, CHOP, C-CASP12 and C-CASP3 from (G) normalized to loading control GAPDH. n=6, ns stands for not significant, *P<0.05, **P<0.01.

To assess changes in ROS levels after SCI, we carried out a time-course study of ROS oxidation products - 8-OHdG, AOPP, and MDA - by ELISA. The levels of 8-OHdG, AOPP, and MDA all peaked at day 1 following injury followed by a gradual decrease by day 7 (Figure 3A). This suggests that accumulation of ROS peaked at day 1 after SCI, which corresponds with the time of peak lysosome dysfunctionally, suggesting a potentially causative relationship between accumulation of ROS and lysosomal malfunction in neurons following SCI. Next, we tested whether ROS accumulation induced lysosomal malfunction and inhibited autophagy flux in neurons after SCI. Pre-treatment of MnTBAP, a ROS scavenger, was administered for SCI and non-SCI mice. Results showed that MnTBAP decreased 8-OHdG, AOPP, and MDA levels in SCI mice on Day1 (Figure 3B), at which time the levels of Beclin1 and VSP34 were all decreased in the SCI + MnTBAP group compared with SCI group (Figure 3C and E). These results suggest that autophagosomes recruitment was inhibited by reduced ROS in SCI.

We also found that MnTBAP enhanced autophagy flux in SCI mice as evidenced by increased levels of LAMP2 and C-CTSD; in addition, both p62 and UB levels were persistently lower (Figure 3C and E). Although the autophagosomal marker, LC3Ⅱ, was downregulated in by MnTBAP (Figure 3C and E), LC3 process assay showed that turnover of LC3Ⅱ in SCI + MnTBAP was likely increased since co-treatment with CQ restored LC3II levels (Figure 3D and F). Immunofluorescence also showed less LC3Ⅱ signals and p62 intensity in neurons following MnTBAP treatment (Figure S3A-C). These results suggest that autophagy flux in SCI mice may be increased with reduced oxidative stress.

Finally, we examined the effect of MnTBAP on ER stress-induced apoptosis levels after SCI. Results revealed that MnTBAP down-regulated the expressions of GRP78, PDI, p-PERK, p-eIF2α, ATF4, CHOP, C-CASP12, and C-CASP3 by day 3 after SCI (Figure 3G and H). In addition, as shown in Figure S3A, D, and E, the intensities of CHOP and CASP3 immunofluorescence staining in motor neurons were also decreased by MnTBAP treatment. Taken together, our data indicate that ROS-induced lysosomal malfunction triggers significant disruptions in autophagy flux, thereby leading to ER stress-induced apoptosis in neurons after SCI.

### Lysosomal dysfunction by ROS leads to autophagy flux blockade and ER stress-induced apoptosis in neural cells

To further investigate the relationship between ROS, autophagy flux, and ER-stress induced apoptosis in neuronal cells, we examined whether ROS accumulation induces lysosomal dysfunction and subsequent autophagy flux blockade and ER stress-induced apoptosis in neural cells *in vitro*. ROS was generated by TBHP treatment to causes oxidative damage via oxidation of membrane lipids and release of lipid peroxidation product MDA 34. Then, we used the fluorescent probe DCFH-DA, a ROS indicator, to detect the intracellular ROS levels in PC12 cells. As expected, TBHP notably increased fluorescence intensity in a dose-dependent manner, and additional treatment of NAC, a ROS scavenger, significantly decreased the fluorescence intensity in high dose TBHP (H-TBHP) group (Figure S4A-B). CCK8 assay was used to measure the cell viability, and results showed that TBHP dose-dependently decreased cell viability, whereas NAC administration increased the viability in the H-TBHP group (Figure S4C). We also performed a mCherry-GFP-LC3 assay. The principle of this assay depends on the PH difference. During the autophagic process, when an autophagosome (pH: neutral) fuses with the lysosome to form the autophagolysosome (pH: acidic), the green fluorescent protein (GFP) will be degraded while the red fluorescent protein (RFP) remains. Consequently, in the merged image, yellow punctates denote autophagosomes while red punctates refer to autolysosomes. As shown in Figure S4D-F), compared with the Control group, the number of both red and green punctates were significantly increased in L-TBHP and M-TBHP group, indicating the enhancement of autophagy flux. Compared with the Control group, the number of red punctate was significantly increased in H-TBHP group, while the number of yellow punctate was decreased, indicating the blockage of autopahgy flux. However, NAC treatment obviously reversed the augment in autophagosomes as well as the reduction in autolysosomes in the H-TBHP group, indicated the alleviation of the disrupted autophagy flux. Immunofluorescence staining for LAMP2 expression was performed to assess lysosomal functions. L-TBHP increased LAMP2 intensity in PC12 cells. M-TBHP group showed no significant changes as compared with the Control group; however, there was a decrease in LAMP2 intensity in PC12 cells treated with H-TBHP. NAC treatment elevated the intensity in H-TBHP groups (Figure S4G-H). These results suggest that moderate levels of ROS activate autophagy in neural cells, however, excess levels block autophagy flux. Furthermore, TBHP notably increased the fluorescence intensities of CHOP and C-CASP3 in a dose-dependent manner, and additional treatment of NAC significantly decreased the intensities of CHOP and C-CASP3 in H-TBHP group, respectively (Figure S4I-L).

To determinate the effect of excess ROS on autophagy and ER stress-induced apoptosis in PC12 cells, groups of Control, H-TBHP, and H-TBHP+NAC were further analyzed. Western blotting revealed that levels of Beclin1, VPS34, P62, and LC3Ⅱ were up-regulated in the H-TBHP group when compared with the Control group, while levels of LAMP2 and C-CTSD were down-regulated; NAC treatment reversed these changes (Figure S4 M-N). Results of LC3 turnover assays showed that the turnover of LC3Ⅱ in the H-TBHP+NAC group after Baf-A1 treatment was more than that in the H-TBHP group following Baf-A1 treatment (Figure S4O-P). Finally, TBHP up-regulated the expressions of GRP78, PDI, p-PERK, p-eIF2α, ATF4, CHOP, C-CASP12, and C-CASP3 in PC12 cells, and NAC treatment reversed these changes. (Figure S4 Q-S). Together, these results further solidify our postulation that that ROS leads to lysosomal dysfunction, eventually resulting in autophagy flux blockade and ER stress-induced apoptosis in neural cells.

### Expressions of the MiTF/TFE family of transcription factors are perturbed in neurons following SCI

**Figure 4:**
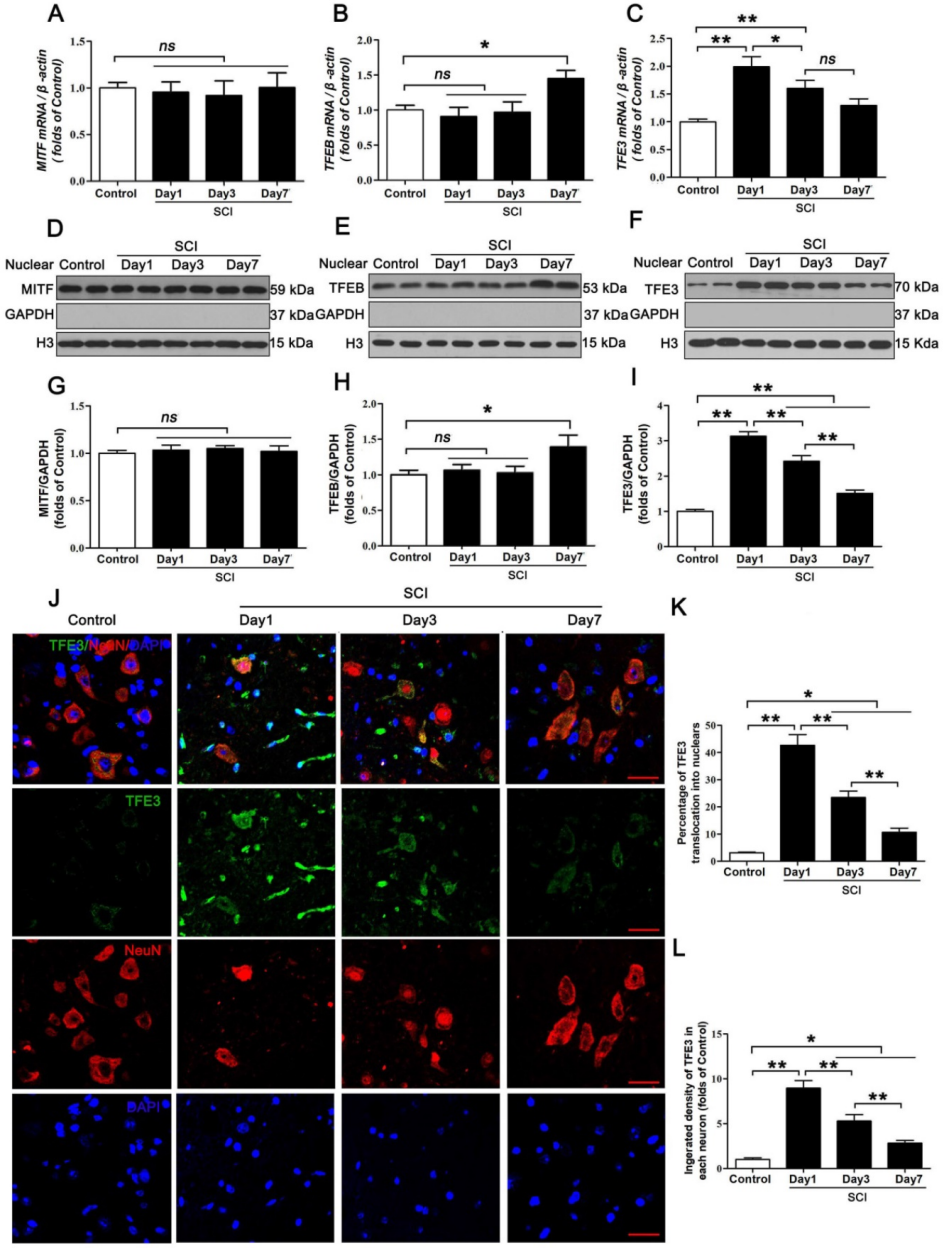
Expressions of the MiTF/TFE family of transcription factors in neurons following SCI. (**A-C**) Relative mRNA level of MiTF/TFE family of transcription factors, including *Mitf*, *Tfeb* and *Tfe3*, in the spinal cord lesions of non-injury (Control) and SCI mice normalized to control *β-actin* at the indicated time points. (**D-F**) Representative Western blotting of MITF, TFEB and TFE3 in Control and SCI mice normalized to loading control GAPDH at specified time points. (**G-I**) Densitometric analysis of MITF, TFEB and TFE3 data from (D-F) normalized to loading control GAPDH. (**J**) Image (30×) of spinal cord sections from the indicated groups stained with antibodies against TFE3/NeuN. Scale bar: 25 µm. (**K-L**) Quantification of immunofluorescence data from (J) showing the percentage of TFE3 nuclear translocation and the mean optical density of TFE3 in spinal cord motor neurons. n=6, ns stands for not significant, *P<0.05, **P<0.01.

The MiTF/TFE family of transcription factors, including MITF, TFEB, TFE3, and TFEC, are global regulators of cell survival and energy metabolism through promotion of lysosomal and anti-oxidative genes 23. Our results showed different time-dependent expression patterns for *Mitf*, *Tfeb,* and *Tfe3* mRNA after SCI. Specifically, there were no changes in the level of *Mitf*, mRNA at days 1, 3 and 7 after injury (Figure 4A); *Tfeb* mRNA level showed no changes at day 1 and 3 after SCI, but was markedly increased at day 7 following SCI (Figure 4B). *Tfe3* mRNA level was significantly up-regulated at day 1 after SCI, then gradually decreased by Day7 (Figure 4C). Western blot analyses of protein expressions of MITF, TFEB, and TFE3 in nuclear extraction of the spinal cord from each group revealed similar trends (Figure 4D-I). Since the expression of TFEC is restricted to cells of myeloid origin 21, we did not investigate TFEC in our study. Of note, peak *Tfe3* mRNA levels and protein expression levels were observed on day 1, which coincides with peak ROS accumulation and lysosomal dysfunction. To further validate these findings, we performed immunofluorescent studies, which showed that immunofluorescence intensity of TFE3 in neurons and the percentage of cells with TFE3-translocated nuclei also peaked at day 1 after injury, gradually decreasing by days 3 and 7 (Figure 4J-L). These results suggest that TFE3 may play a substantial role in ROS-induced lysosomal dysfunction after SCI.

### Reduced expression of TFE3 abates autophagic flux and aggravates ER stress-induced apoptosis in neurons after SCI

**Figure 5:**
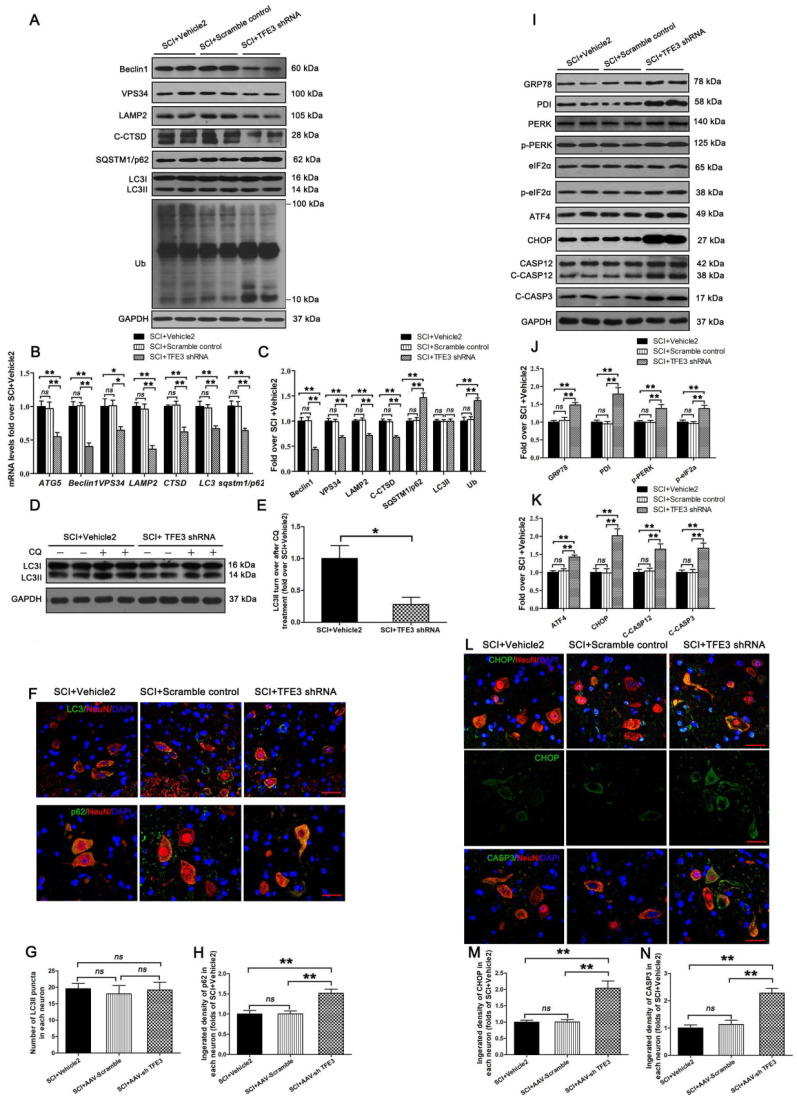
Reduced expression of TFE3 abates autophagy flux and subsequently aggravates ER stress-induced apoptosis after SCI. (**A**) Western blot analysis of autophagy flux markers as indicated in spinal cord lesions from SCI+Vehicle2 mice, and mice injected with AAV-Scramble or AAV-shTFE3, then subjected with SCI, at Day 3. (**B**) Relative mRNA level of *Atg5*, *Beclin1*, *Vps34*, *Lamp2*, *Ctsd*, *Lc3* and *Sqstm1/p62* in the lesions of the indicated groups normalized to control *β-actin* at Day 3. (**C**) Corresponding densitometric analysis of the bands from (A) normalized to the loading control GAPDH. (**D**) Western blotting of LC3II in SCI+Vehicle2 and SCI+AAV-shTFE3 spinal cord slides cultured in the presence or absence of CQ at Day 3. (**E**) Densitometric analysis of LC3II from (D) with respect to the loading control GAPDH. (**F**) Image (30×) of spinal cord sections from the indicated groups at Day3 stained with antibodies against LC3/NeuN and p62/NeuN; scale bar: 25 µm. (**G-H**) Quantification of immunofluorescence data from (F) showing the mean number of LC3II and optical density of p62 in motor neurons at the spinal cord. (**I**) Western blot analysis of ER stress-induced apoptosis markers as indicated in the grouped mice at Day 3 after SCI. (**J, K**) Corresponding densitometric analysis of the bands from (I) normalized to the loading control GAPDH. (**L**) Image (30×) of spinal cord sections from the indicated groups at Day 3 stained with antibodies against CHOP/NeuN and CASP3/NeuN; scale bar: 25 µm. (**M, N**) Quantification of immunofluorescence data from (L) showing the mean optical density of CHOP and CASP3 in motor neurons at the lesion. n=6, ns stands for not significant, *P<0.05, **P<0.01.

To assess the impact of TFE3 expression on autophagic flux and ER stress-induced apoptosis in neurons, we used a TFE3 shRNA AAV vector to knock down TFE3. Indeed, AAV-TFE3 shRNA injection significantly decreased TFE3 level in cell nuclei relative to the SCI + Vehicle2 group (Figure S5A and B). Immunofluorescence showed that AAV-TFE3 shRNA injection was indeed able to down-regulate TFE3 expression in neurons after SCI (Figure S5C and D). Next, we explored if TFE3 inhibition can lead to aggravation of autophagic flux disruption and ER stress-induced apoptosis. Transcriptional levels of autophagy genes targeted by TFE3 - *Atg5*, *Beclin1*, *Vps34*, *Lamp2*, *Ctsb*, *Lc3,* and *Sqstm1/p62* - were measured by qPCR. Results showed that target gene levels were lower in the SCI + TFE3 shRNA group compared with the SCI + Vehicle2 group (Figure 5B). As shown in Figure 5A and C, Beclin1, VPS34, LAMP2, and C-CTSD expression levels were lower in the SCI + TFE3 shRNA group compared with the SCI + Vehicle2 group, with higher expression levels of p62 and UB; AAV-TFE3 shRNA injection did not change LC3 protein level in SCI mice. LC3 turnover assay indicated that turnover of LC3Ⅱ in SCI+TFE3 shRNA mice after CQ treatment was slower than in SCI + Vehicle2 mice following the use of CQ (Figure 5D-E). Immunofluorescence showed increased p62 intensity in neurons in SCI+TFE3 shRNA mice compared with SCI+ Vehicle2 mice, but the number of LC3Ⅱ dots was not significantly different between the two groups (Figure 5F-H). Similar results were found between SCI+TFE3 shRNA and Scramble control groups in the above tests, and there was no significant difference between the SCI + Vehicle2 and SCI + Scramble control groups. Together, these data suggest that autophagy flux disruption in neurons after SCI can be directly induced by the transcriptional depression of autophagic genes targeted by TFE3, highlighting the critical role of TFE3 in the regulation of autophagy flux in SCI.

As shown in Figure S5E-G, we found that both transcriptional and protein levels of anti-oxidation markers targeted by TFE3, SOD1, HO1, PGC1α, TRX, and GPX3 were down-regulated in SCI + TFE3 shRNA mice compared with SCI+ Vehicle2 mice. Meanwhile, a similar expression pattern of SOD1 was observed in neurons from both groups (Figure S5H and I). ELISA results revealed that levels of 8-OHdG, AOPP, and MDA were higher in SCI + TFE3 shRNA mice than SCI + Vehicle2 mice (Figure S5J-L). Similar results were found between SCI+TFE3 shRNA and Scramble control groups in the above tests, with no significant differences between the SCI + Vehicle2 and SCI + Scramble control groups. These data indicated that autophagy flux disruption in neurons after SCI can be induced by the transcriptional depression of autophagy and anti-oxidation genes targeted by TFE3.

Western blotting revealed that expressions of GRP78, PDI, p-PERK, p-eIF2α, ATF4, C-CASP12, and C-CASP3 in SCI + TFE3 shRNA mice were significantly greater than in SCI + Vehicle2 mice (Figure 5I-K). Similar expression patterns of CHOP and CASP3 were observed in neurons from these three groups (Figure 5L-N). As expected, the SCI+TFE3 shRNA group showed a larger area of glial scar (Figure S6A-B), less number of ventral motor neurons (Figure S6C-D), decreased MAP2 expression (Figure S6E-F), and down-regulated SYN positive synapses in ventral motor neurons (Figure S6G-H). In addition, compared with the SCI+Vehicle2 group, BMS scores were significantly decreased in SCI+TFE3 shRNA mice 7, 21 and 28, days after SCI (Figure S6J). Footprint analyses indicated that SCI + TFE3 shRNA mice had worse restoration of hind legs movement with coordinated crawling compared with the SCI+ Vehicle2 group (Figure S6K). Similar results were found when comparing SCI+TFE3 shRNA and Scramble control groups in the above tests, with no significant differences between the SCI + Vehicle2 and SCI + Scramble control groups. Overall, these sets of experiments indicate that reduced expression of TFE3 abates autophagy flux, aggravates ER stress-induced apoptosis in neurons, and inhibits functional recovery after SCI.

### Overexpression of TFE3 augments autophagy flux and ameliorates ER stress-induced apoptosis in neurons following SCI

**Figure 6:**
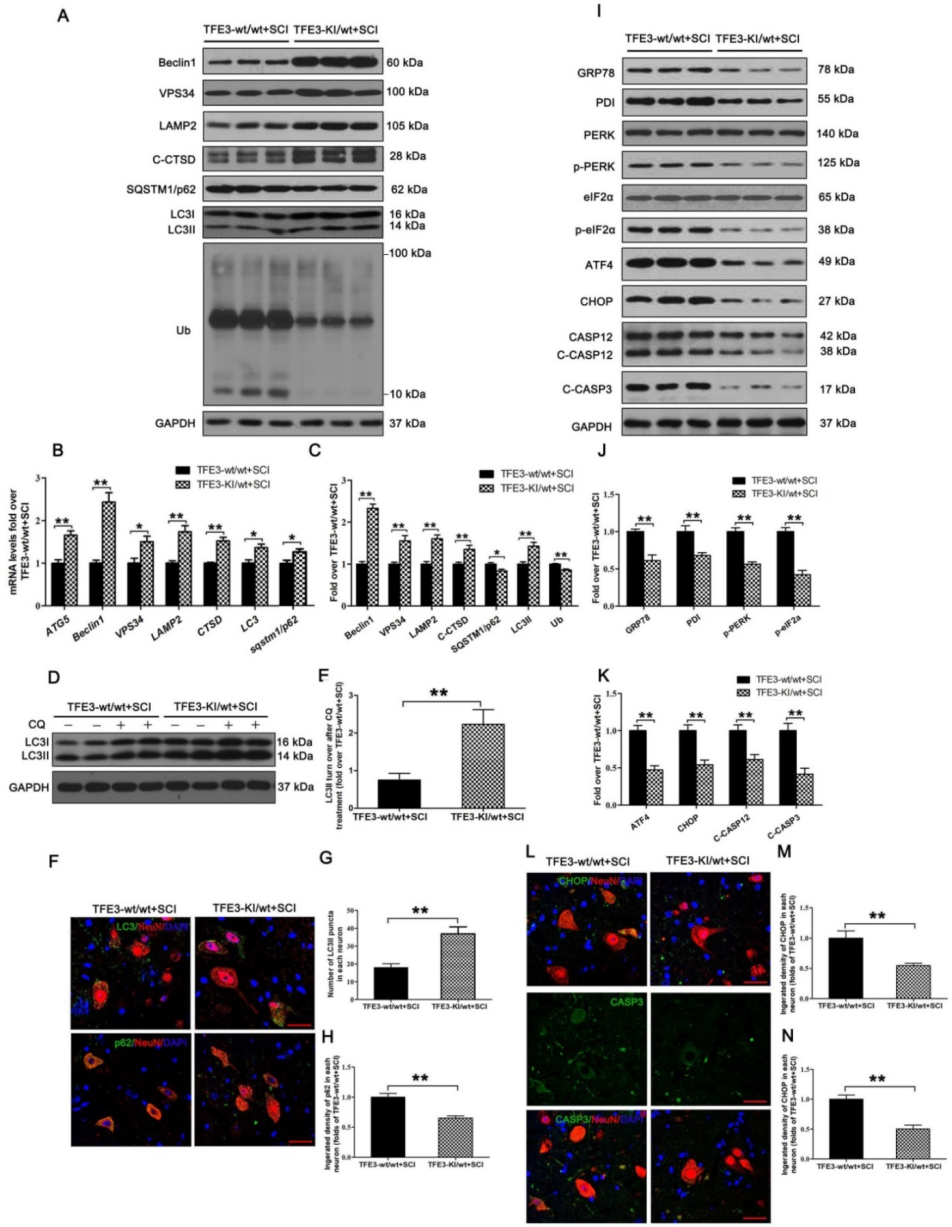
Overexpression of TFE3 augments autophagy flux and subsequently ameliorates ER stress-induced apoptosis after SCI. (**A**) Western blot analysis of autophagy flux markers as indicated in the spinal cord lesion from TFE3-KI/wt mice and TFE3-wt/wt mice at Day 3 after SCI. (**B**) Relative mRNA level of *Atg5*, *Beclin1*, *Vps34*, *Lamp2*, *Ctsd*, *Lc3* and *Sqstm1/p62* in the lesions of both groups normalized to control *β-actin* at Day 3. (**C**) Densitometric analysis of band data from (A) with normalized to the loading control GAPDH. (**D**) Western blotting of LC3II in the indicated mice spinal cord slides cultured in the presence or absence of CQ at Day 3. (**E**) Densitometric analysis of LC3II from (D) with respect to the loading control GAPDH. (**F**) Image (30×) of spinal cord sections from the indicated groups at Day 3 stained with antibodies against LC3/NeuN and p62/NeuN; scale bar: 25 µm. (**G-H**) Quantification of immunofluorescence from (F) showing the mean number of LC3II and optical density of p62 in motor neurons at the spinal cord. (**I**) Representative Western blotting for ER stress-induced apoptosis markers as indicated in the both groups, at Day3 after SCI. (**J, K**) Densitometric analysis of the data from (I) normalized to loading control GAPDH. (**L**) Image (30×) of spinal cord sections from the both groups stained with antibodies against CHOP/NeuN and CASP3/NeuN; scale bar: 25 µm. (**M, N**) Quantification of data from (L) indicating the mean optical density of CHOP and CASP3 in motor neurons at the lesion. n=6, *P<0.05, **P<0.01.

TFE3-KI/wt mice, which specifically over-expressing TFE3, and their corresponding wide type mice TFE3-wt/wt mice were used to determine the effect of increased TFE3 expression. Indeed, at day 3 after SCI, levels of TFE3 nuclear translocation in TFE3-KI/wt mice were much higher than TFE3-wt/wt mice (Figure S7A-D). Furthermore, qPCR demonstrated that transcriptional levels of *Atg5*, *Beclin1*, *Vps34*, *Lamp2*, *Ctsb*, *Lc3,* and *Sqstm1/p62* were higher in TFE3-KI/wt +SCI mice than in TFE3-wt/wt + SCI mice (Figure 6B); similar trends were seen for the protein levels of autophagy-related markers VPS34, LAMP2, C-CTSD and LC3Ⅱ(Figure 6A and C). LC3 turnover assay showed that turnover of LC3Ⅱ in TFE3-KI/wt + SCI mice after CQ treatment was more than that in TFE3-wt/wt + SCI mice following the using of CQ (Figure 6D-E). Immunofluorescence indicated that a greater number of LC3Ⅱ dots and less p62 intensity co-localization with neurons in TFE3-KI/wt +SCI mice (Figure 6F-H). Overall, these findings suggest that autophagy flux disruption in neurons after SCI can be directly alleviated by overexpression of TFE3.

As expected, transcriptional and expressional levels of anti-oxidation markers targeted by TFE3 - SOD1, HO1, PGC1α, TRX, and GPX3 - were increased in TFE3-KI/wt +SCI mice (Figure S7E-G), and a similar expression pattern of SOD1 was observed in neurons from both groups (Figure S7H and I). ELISA also demonstrated that levels of 8-OHdG, AOPP, and MDA were lower in TFE3-KI/wt + SCI mice at day 3 (Figure S7J-L). Together, these data indicate autophagy flux disruption in neurons after SCI can be alleviated by the transcriptional promotions of autophagy and anti-oxidation genes targeted by TFE3.

Western blotting showed that protein levels of PDI, p-PERK, p-eIF2α, ATF4, C-CASP12, and C-CASP3 in TFE3-KI/wt + SCI mice were significantly reduced compared to TFE3-wt/wt + SCI mice (Figure 6I-K). Similar expression patterns of CHOP and CASP3 were observed in neurons from both groups (Figure 6L-N). As expected, TFE3-KI/wt + SCI mice showed a smaller area of glial scar (Figure S8A-D), a higher number of ventral motor neurons (Figure S8C-D), increased MAP2 expression in neurons (Figure S8E-F), and increased synapse onto ventral motor neurons (Figure S8G-H). Analysis of survival rate revealed that there no significant difference between TFE3-KI/wt +SCI group and TFE3-wt/wt + SCI group (Figure S8I). BMS scores were significantly increased in TFE3-KI/wt + SCI after SCI at days 21 and 28, respectively, when compared with TFE3-wt/wt + SCI mice (Figure S8J). Finally, footprint analyses showed that at day 28 after injury, TFE3-KI/wt +SCI mice showed a better restoration of hind legs movement with coordinated crawling than TFE3-wt/wt + SCI mice (Figure S6K). Overall, these results suggest that overexpression of TFE3 enhances autophagy flux, alleviates ER stress-induced apoptosis in neurons, and promotes functional recovery after SCI.

### The activity of TFE3 after SCI is regulated by AMPK-mTOR and AMPK-SKP2-CARM1 signaling pathways

**Figure 7:**
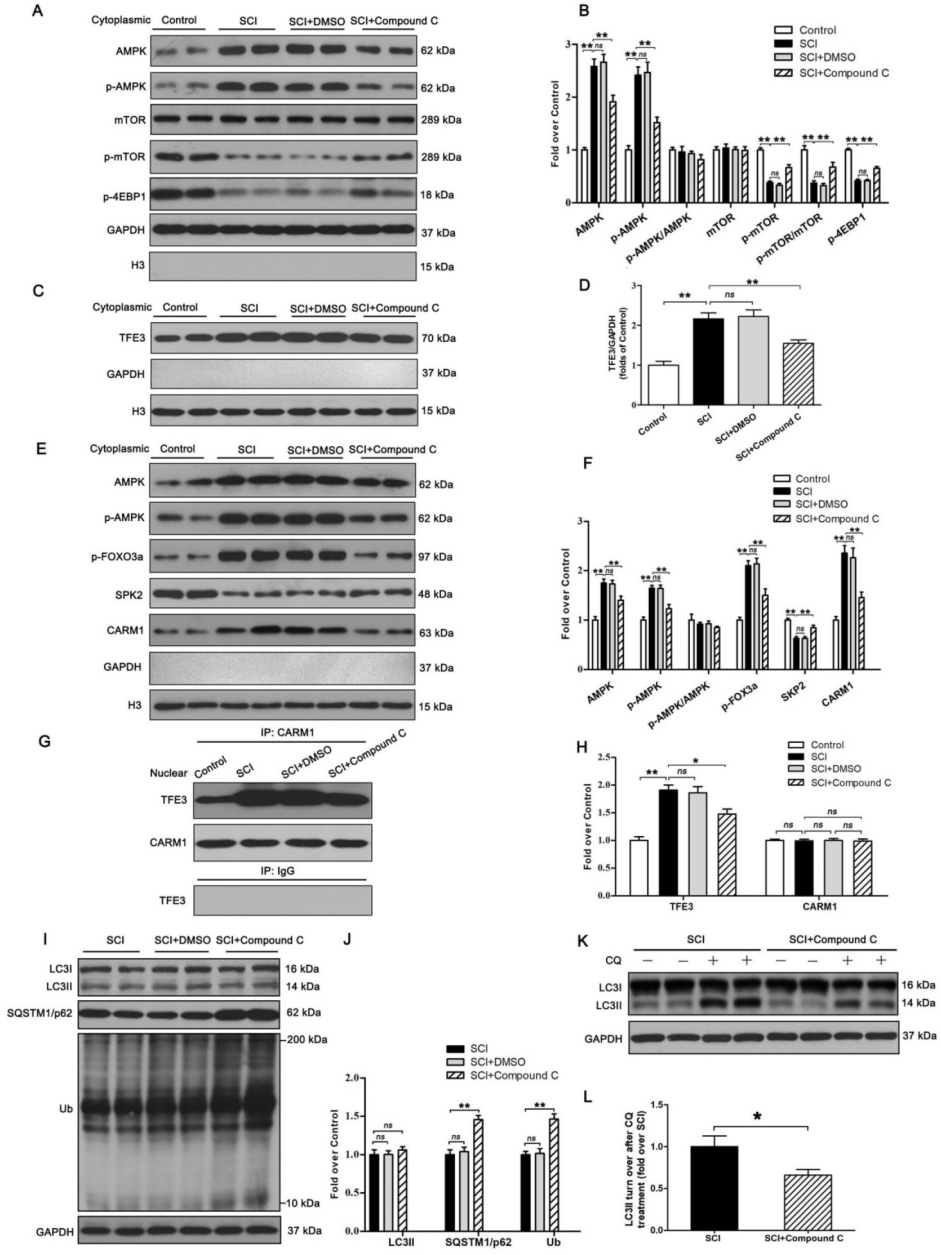
The activity of TFE3 after SCI is regulated by AMPK-mTOR and AMPK-SKP2-CARM1 signaling pathways. (**A**) Western blot analysis of AMPK-mTOR signal pathway in the cytoplasm in spinal cords from the control mice, SCI mice, and SCI mice treated with DMSO or Compound C, at Day3. (**B**) Densitometric analysis of the AMPK, p-AMPK, p-mTOR and p-4EBP1 data from (A), normalized to the loading control GAPDH. (**C**) Western blotting of TFE3 nuclear translocation at the lesion for each group. (**D**) Corresponding densitometric analysis of the TFE3 bands in (C) normalized to the loading control H3. (**E**) Western blots of the AMPK-SKP2-CARM1 signaling pathway in the nucleus of the indicated groups at Day3 after SCI. (**F**) Densitometric analysis of AMPK, p-AMPK, p-FOXO3a, SKP2 and CARM1 bands from (E) normalized to control H3. (**G**) Nuclear CARM1-TFE3 complex was detected by IP in indicated groups at Day3 after SCI. (**H**) Densitometric analysis of TFE3 and CARM1 data from (G) normalized to loading control H3. (**I**) Western blot analysis of LC3, SQSTM1/p62 and UB in the spinal cord lesion of each group at Day3 after SCI. (**J**) Densitometric analysis of band data from (I) normalized to the loading control GAPDH. (**K**) Western blotting of LC3II in the indicated mice spinal cord slides cultured in the presence or absence of CQ at Day3. (**L**) Densitometric analysis of LC3II from (K) normalized to the loading control GAPDH. n=6, ns stands for not significant, *P<0.05, **P<0.01.

Finally, we sought to investigate the mechanism by which TFE3 activation and nuclear translocation were increased following SCI. Here, we focused on AMPK, a key responder to starvation and low-energy states that has been shown to be a key regulator in the activities of MiTF/TFE family. The nuclear translocation of MiTF/TFE family was closely associated with the activation of the AMPK-mTOR signaling pathway in the cytoplasm. Western blotting of cytoplasmic proteins revealed that the level of phosphorylated AMPK in the SCI group was increased relative to the Control group, while phosphorylated levels of mTOR and 4EBP1 were decreased and the ratios of p-AMPK/AMPK and p-mTOR/mTOR were unchanged (Figure 7A-B). In the nucleus, the activation of AMPK-FoxO3a-SKP2 signaling cascade increases the level of CARM1, which binds to TFEB and methylates promoter sequences to induce efficient transcription. TFE3 and TFEB often share regulatory signaling networks, so we postulated that the activity of TFE3 in nuclear maybe regulated by AMPK-SKP2-CARM1 signaling. As shown in Figure 7E-F, Western blot analysis of nuclear proteins revealed that AMPK, p-AMPK, p-FOXO3a, and CARM1 were increased in the SCI group compared with the control group. SKP2 was decreased in the SCI group compared with the control group, while the ratio of p-AMPK/AMPK was unchanged. Furthermore, IP analysis for far Western blotting showed that the level of CARM1 and TFE3 binding was increased in the spinal cord after injury (Figure 7G-H). These results indicate that increased activity of TFE3 after SCI may be regulated by activating the AMPK-mTOR and AMPK-SKP2-CARM1 signaling pathways.

To further determine the relationship between TFE3 activity and the AMPK-mTOR and AMPK-SKP2-CARM1 signaling pathways after SCI, Compound C, a small-molecule cell-permeable pyrazolopyrimidine derivative that acts as a reversible ATP-competitive inhibitor of AMPK 35, and its solvent (DMSO) were administrated for SCI mice. Both Western blotting and IP showed that Compound C significantly inhibited the AMPK-mTOR and AMPK-SKP2-CARM1 pathways in the spinal cord following injury, and also decreased the levels of TFE3 nuclear translocation, and CARM1 and TFE3 binding (Figure 7A-H). Together, these results demonstrate that the activity of TFE3 after SCI is partly regulated by AMPK-mTOR and AMPK-SKP2-CARM1 signaling pathways.

**Figure 8:**
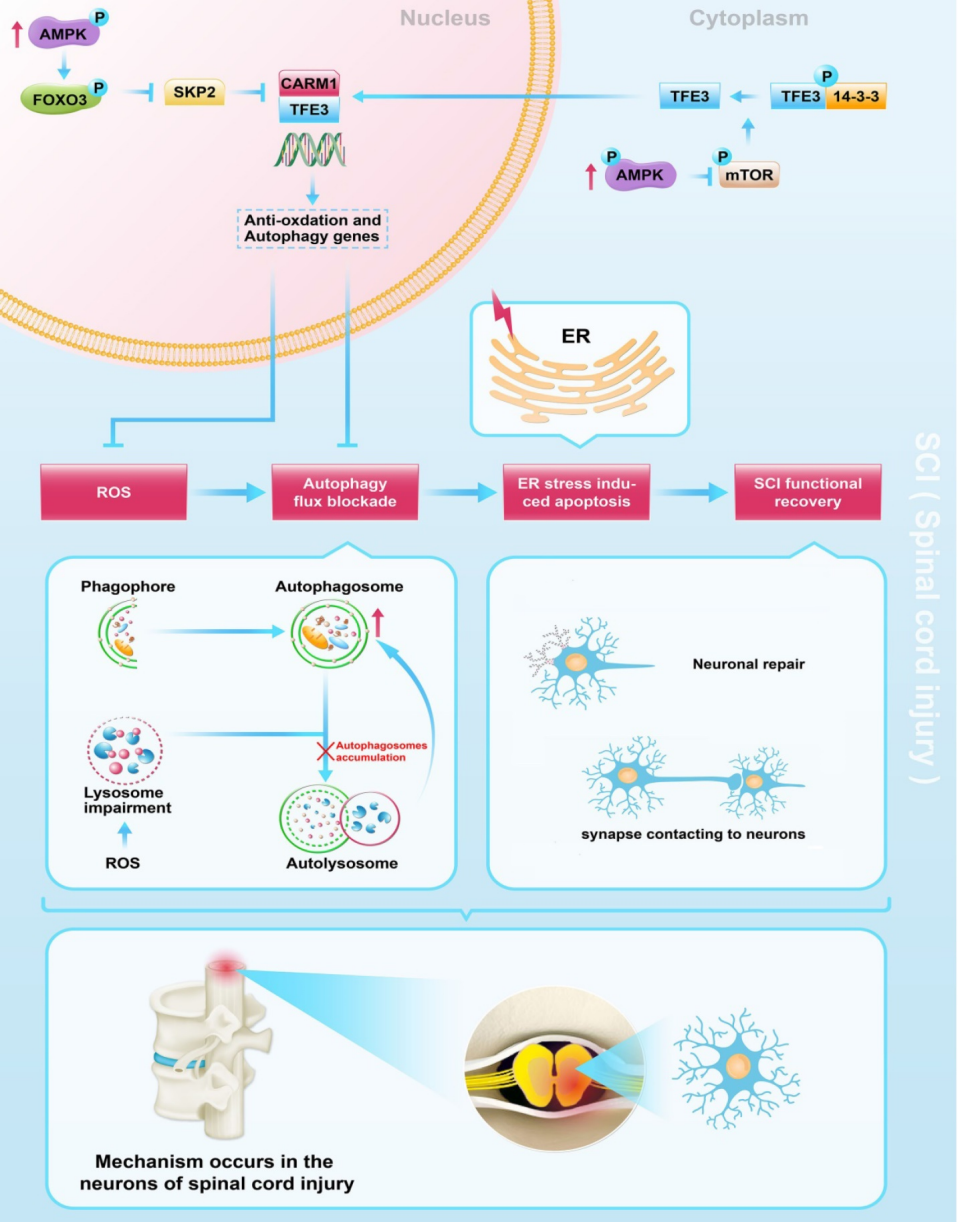
Schematic illustration of the proposed molecular mechanism highlighting the role of TFE3, ROS, and autophagy in the pathophysiology of SCI and subsequent neurological recovery.

Autophagy flux after Compound C treatment in SCI mice was evaluated as well, and as shown in Figure 8I-L, SQSTM1/p62, and UB expression levels were higher in the SCI+Compound C when compared to the SCI group. Compound C treatment did not change the LC3 protein level in SCI mice. LC3 turnover assay indicated that turnover of LC3Ⅱ in SCI+Compound C mice after CQ treatment was slower than that in SCI mice following the use of CQ (Figure 8K-L). These results indicate that Compound C treatment inhibit autophagy flux after SCI.

## Discussion

This study provides novel evidence that TFE3 is an important regulator in ROS-mediated autophagy dysfunction following SCI. We found that TFE3 directly impacts ROS levels in SCI and that ROS induced by SCI is responsible for lysosomal dysfunction, autophagy flux blockade, and subsequent ER-stress mediated neuronal apoptosis. Furthermore, we found that AMPK-mTOR and AMPK-SKP2-CARM1 signaling pathways may play a critical role in SCI-induced activation of TFE3. More importantly, we showed that modulations of TFE3 levels result in profound impacts on the outcomes of SCI in our animal model, suggesting that TFE3 may be a promising therapeutic target for SCI.

Autophagy is defined as a highly regulated process involved in the turnover of long-lived proteins, cytosolic components, or damaged organelles 36. Autophagy generates energy through a lysosome-dependent degeneration pathway for cells under nutrient-poor conditions or during starvation to maintain cellular homeostasis 37. It has been reported that impairment of autophagy was implicated in neurodegenerative disorders such as Parkinson's and Alzheimer's diseases 38. However, few studies paid attention to the impaired autophagy flux and its functional definition in the field of SCI. In the current study we found that the initiation process of autophagosome was significantly activated in neurons on day 1 after injury, and then gradually decreased by day 7. Lysosomal function was impaired in neurons on day 1 after SCI, then returned to baseline levels on day3, and significantly increased on day 7, without transcriptional changes in genes responsible for lysosomal biogenesis. Moreover, accumulation of substrate proteins, p62 and UB, in neurons was increased on days 1 and 3, remaining elevated on day 7. These data suggest that autophagy flux is disrupted in neurons after SCI, with disturbances peaking at day 1. Our result is consistent with Liu et al.'s findings 3. Regarding the controversy of autophagy flux situation after SCI 39, 40, our work provides valuable evidence that autophagy impairment occurs following SCI.

ER stress is known to induce and promote apoptosis during secondary injury following SCI 41. Importantly, ER stress was found to be closely related to the process of autophagy and can induce autophagy in mammalian cells, with several canonical UPR pathways implicated in the interaction 14, 15. Here, we hypothesized that disrupted autophagy flux may potentiate ER stress and induce apoptosis in neurons after SCI. Our results demonstrate that ER stress-induced apoptosis peaked at day 3, a time point following autophagy flux blockade at day 1. In addition, ATG5^-/+^ mice showed more aggravated autophagy flux inhibition, increased ER stress-induced apoptosis in neurons, and deterioration of functional recovery after SCI. To our knowledge, we were the first to utilize transgenic mice to uncover that blockade of autophagy flux exacerbates ER stress-induced apoptosis in neurons after SCI. This result implies that autophagy may be beneficial in preventing cell death and promoting neurological repair following spinal cord injury.

Bcl-2 family proteins such as BAX, chaperone heat shock protein HSP70, and ROS, lead to lysosomal membrane destabilization 42, 43, 44 and are considered to be three potential factors contributing to altered autophagy flux after CNS trauma 2. However, few studies focus on the mechanisms on lysosome-induced autophagy disruption after SCI. In our study, the accumulation of ROS peaked at day 1 after SCI, a time point that coincides with maximum lysosome dysfunctionality, suggesting a potentially causative relationship between accumulation of ROS and lysosomal malfunction in neurons following SCI. In our *in vitro* experiments, it was also revealed that excess ROS disrupted lysosomal and autophagy functions, which was relieved by ROS scavenger, NAC. Furthermore, in SCI mice, anti-oxidant therapy alleviated disruptions to autophagy. Thus, ROS-induced lysosomal malfunction is the culprit for disruptions of autophagy flux in neurons after SCI.

The MiT/TFE family, including MITF, TFEB, TFE3, and TFEC, plays crucial roles in the regulation of lysosomal function and oxidative metabolism 45, 46, however, its involvement in SCI is poorly defined. Under conditions with high autophagy demand, the MiT/TFE family coordinates an efficient transcriptional program to up-regulate genes responsible for autophagic flux enhancement 47. The MiT/TFE family also reduces intracellular ROS by increasing the abundance of antioxidant genes 24. Interestingly, both TFEB and TFE3 were recently identified as potential therapeutic targets to rescue neurological diseases 48, 49, 50, 51. Our results revealed that TFE3 mRNA levels and protein expression in neurons peak on day 1 after SCI. This time point coincides with maximal ROS accumulation and lysosomal dysfunction, suggesting TFE3 may play a substantial role in the amelioration of ROS-induced lysosomal malfunction. Our AAV-sh TFE3 experiments showed that reduced TFE3 leads to decreased autophagy flux and subsequent ER stress-induced apoptosis in neurons, ultimately impeding functional recovery after SCI. There are dozens of Adeno-associated viruses (AAVs) with different capsid protein spatial structures, sequences, and tissue specificities. Among the AAVs, AAV9 is the most commonly used because of its wide infection spectrum in organs such as the brain, spinal cord, lung, liver, muscle, and heart. We went on to test whether overexpression of TFE3 promotes autophagic flux by generating a TFE3 knock-in transgenic allele with CRISPR. We observed enhanced autophagic flux in TFE3-KI/wt mice, confirming the effect of TFE3 in promoting autophagic flux. Notably, when present in the homozygous state, TFE3-KI allele causes lethality, which might be due to CRISPR off-target mutations. Although we are unable to rule out the possibility that the increased autophagic flux in TFE3-KI/wt is caused by an unknown non-specific off-target mutation, the opposite effects between shTFE3 and TFE3-KI suggest that the effect on autophagic flux is TFE3-specific.

Thus, to our knowledge, we are the first to demonstrate that TFE3 ameliorates autophagy flux blockade via the promotion of autophagy-lysosomal activity and depression of oxidative stress, subsequently leading to alleviation of ER stress-induced apoptosis in neurons and promotion of functional recovery after SCI. Interestingly, several studies have shown that trehalose, a pharmacological agonist of TFE3 52, exerts neuroprotective effects following spinal cord injury in rats 53, 54. However, whether its effects are due to TFE3 mediated autophagy enhancement is unknown. Thus, future evaluations of pharmacological TFE3 agonists such as trehalose should be investigated in the context of autophagy in SCI.

We also conducted studies to investigate upstream signals of TFE3 upregulation in SCI. AMPK, a responder to starvation and low-energy states, has been shown to be a key regulator in the activities of MiT/TFE family 24, and previous data suggest that nuclear translocation of TFE3 is closely associated with the activation of AMPK-mTOR signaling pathway in the cytoplasm 55. The mTOR mediated dephosphorylation of Ser 321 of TFE3, leads to the dissociation of the TFE3-14-3-3 complex, allowing free TFE3 to enter the nucleus 56. AMPK is also involved in modulating MiT/TFE family transcription factor activity through SKP2-CARM1 signaling 28. In the nucleus, the activation of AMPK-FoxO3a-SKP2 signaling cascade increases the level of CARM1, a co-activator of TFEB, which binds to TFEB and methylates promoter sequences, thereby inducing efficient transcription of intracellular autophagic and anti-oxidative genes 29. TFE3 and TFEB were found to often share regulatory signaling networks 52, thus, it is possible that TFE3 in the nucleus may also be regulated by AMPK-SKP2-CARM1 signaling. To our knowledge, our results are the first to report that activation of TFE3 after SCI is partly regulated by AMPK-mTOR and AMPK-SKP2-CARM1 signaling pathways. Of note, recent studies found that TFE3 dephosphorylation and translocation of TFE3 into the nucleus may be triggered by increased intracellular Ca^2+^ levels and PPP3/calcineurin activation 57, 58. Thus, it is possible that TFE3 activity may be also regulated by the Ca^2+^-PPP3/calcineurin signaling pathway. Furthermore, as very few studies have investigated the role of non-coding RNAs in autophagy following SCI, investigations on whether microRNA (miRNA) or long non-coding RNAs (lncRNAs) affect TFE3 activity in SCI is also of great importance.

While the current study focused on ER-stress induced neuronal apoptosis, other types of programmed cell death such as necroptosis, ferroptosis, and pyroptosis can also be affected by autophagic and lysosomal mechanisms. Therefore, our findings regarding TFE3, ROS, and autophagy also applies to necroptosis, ferroptosis, and pyroptosis in SCI should be further verified.

In conclusion, we found that SCI leads to an excessive accumulation of ROS, results in lysosomal malfunction, blocks autophagy flux, induces ER stress-induced apoptosis in neurons, and ultimately leads to poor outcomes following SCI. Furthermore, we uncovered that in SCI, TFE3 plays a critical role in regulating these processes (Figure 8), and maybe a promising therapeutic target for the future treatments of SCI.

## Material and Methods

### Reagents and antibodies

Chloroquine (C18H26ClN3 · 2H3PO4, HPLC > 98%, Cat. No. C6628), MnTBAP (C96H56Cl2Mn2N8O16, HPLC > 98%, Cat. No. 475870), Dimethyl sulfoxide (CH3)2SO, HPLC > 99.7%, Cat. No. D2650), Bafilomycin A1 (C70H116O18, HPLC ≥97%, Cat. No.196000) and IGEPAL CA-630 (Cat. No. I8896) were purchased from Sigma-Aldrich (St. Louis, MO, USA). Dorsomorphin (Compound C, C24H25N5O; purity≥ 98.14%, Cat. No. 866405-64-3) was acquired from Med Chem Express (Monmouth Junction, NJ, USA). N-Acetyl-L-Cysteine (HSCH2CH (NHCOCH3) CO2H, HPLC > 98%, Cat. No. 616-91-1), HE staining kit (Cat. No. G1120), Masson staining kit (G1340), and Nissl staining kit (Cat. No. G1430) were obtained from Solarbio Science & Technology (Beijing, China). 2',7'-dichlorodihydrofluorescein diacetate (DCFH-DA, Cat. No. S0033) and Ad-mCherry-GFP-LC3B adenovirus (Cat. No. C3011) were acquired from Beyotime Institute of Biotechnology (Shanghai, China). AAV-TFE3 shRNA adeno-associated virus (serotype 9, without a fluorescent reporter gene) was designed by GeneChem Chemical Technology Co., Ltd (Shanghai, China). Primary antibody against TFEB was purchased from Bethyl Laboratories (A303-673A-M; Montgomery, TX, USA). Primary antibodies against Beclin1 (Cat. No. 3738), ATG5 (Cat. No. 12994), ATP6V1B2 (Cat. No. 14617), Ubiquitin (Cat. No. 3936), ATF4 (Cat. No. 11815), CHOP (Cat. No. 2895), AMPKα (Cat. No. 5832), p-AMPKα (Cat. No. 2535) p-FOXO3a (Cat. No. 9466), p-EIF2α (Cat. No. 3398), p-4EBP1 (Cat. No. 9456), mTOR (Cat. No.2983), p-mTOR (Cat. No.5536) and CARM1 (Cat. No. 4438) were purchased from Cell Signaling Technology (Beverly, MA, USA). VPS34 (Cat. No. 12452-1-AP), CTSD (Cat. No. 21327-1-AP), MITF (Cat. No. 13092-1-AP), SOD1 (Cat. No. 10269-1-AP), HO1 (Cat. No. 10701-1-AP), Thioredoxin (Cat. No. 14999-1-AP), GPX3 (Cat. No. 13947-1-AP), SKP2 (Cat. No. 15010-1-AP), eIF2α (Cat. No. 11233-1-AP), Histone-H3 (Cat. No. 17168-1-AP) and GAPDH (Cat. No. 10494-1-AP) were obtained from Proteintech Group (Chicago, IL, USA). LAMP2 (Cat. No. ab13524), p62/SQSTM1 (Cat. No. ab56416), GRP78 (Cat. No. ab21685), PDI (Cat. No. ab2792), NeuN (Cat. No. ab177487), CASP3 (Cat. No. ab13847), CASP12 (Cat. No. ab62484), Synaptophysin (Cat. No. ab32594), MAP2 (Cat. No. ab32454), PCG1α (Cat. No. ab54481), goat anti-rabbit IgG H&L (Alexa Fluor® 488) (Cat. No. ab150077), and goat anti-mouse IgG H&L (Alexa Fluor® 647) (Cat. No. ab150115) were obtained from Abcam (Cambridge, UK). Primary antibody against TFE3 (Cat. No. HPA023881) was purchased from Sigma-Aldrich Chemical Company (Milwaukee, WI, USA). PERK (Cat. No. BS2156), goat anti-mouse IgG (H+L) -HRP (Cat. No. BS12478) and goat anti-rabbit IgG (H+L)-HRP (Cat. No. BS13278) were acquired from Bioworld Technology (Minneapolis, MN, USA). p-PERK (Cat. No. Orb336657) was obtained from Biorbyt Company (Cambridge, UK). LC3B (Cat. No. NB600) was purchased from Novus Biologicals (Littleton, CO, USA). Cleaved CASP3 (Cat. No. AF7022) was purchased from Affinity (Cincinnati, OH, USA). Pierce Crosslink immunoprecipitation kit (Cat. No. abab26147) was purchased from Thermo Scientific (Madison, WI, USA). Fetal bovine serum (Catalog No. p30-3301) was obtained from PAN-Biotech GmbH (Aidenbach, Germany). Tert-butyl hydroperoxide solution (TBHP, Catalog No. 458139) was purchased from Merck (Darmstadt, Germany). TUNEL apoptosis detection kit (40307ES20) was obtained from Yeasen Biochemical (Shanghai, China).

### Production of ATG5^-/+^ mice

ATG5^-/+^ mice were provided by Suzhou Saiye Biotechnology Co., Ltd. The mouse ATG5 gene (GenBank accession number: NM_053069.5; Ensembl: ENSMUSG00000038160) is located on mouse chromosome 10. Exon 3 was selected as the target site. TALEN mRNAs generated by *in vitro* transcription were then injected into fertilized eggs for KO mouse productions. The founders were genotyped by PCR followed by DNA sequencing analysis. The positive founders were breeding to the next generation which was genotyped by PCR and DNA sequencing analysis. ATG5 homozygous knockout (ATG5^-/-^) is lethal. So ATG5 hemi-zygote knockout (ATG5^-/+^) mice were selected for our experiments.

### Production of TFE3-KI/wt mice

TFE3-KI/wt mice were produced by Nanjing Biomedical Research Institute of Nanjing University. Transcript Tfe3-201 (ENSMUST00000077680.9) is selected for the production of TFE3-KI/wt mice. Tfe3-201 gene has 10 exons, with the ATG start codon in exon 1 and TGA stop codon in exon 10. H11-CAG-Tfe3-flag-polyA knockin mice were made via the CRISPR/Cas9 system. Cas9 mRNA, sgRNA, and donor were co-injected into zygotes, sgRNA directed Cas9 endonuclease cleavage at H11 locus, and created a DSB (double-strand break). Such breaks will be repaired, and result in CAG-Tfe3-flag-polyA inserted in H11 locus. The pups will be genotyped by PCR, followed by sequence and southern blot analysis. TFE3 homozygous knockin (TFE3-KI/KI) is lethal, so, TFE3 hemi-zygote knockin (TFE3-KI/wt) mice were selected for our experiments.

### Animals, groups, and treatments

All experimental protocols were approved by Wenzhou Medical University's Animal Research Committee (wydw 2017-096). All animals used in the study were cared for in accordance with the ethical guidelines on animal experimentation of Laboratory Animals of China National Institutes of Health. Healthy female C57BL/6 mice (weight 20~30g) were obtained from the Experimental Animal Center (License no. SCXK 2005-0019) of Wenzhou Medical University, Zhejiang Province, China. C57BL/6 mice were randomly divided into the Control group, SCI-Day 1 group (SCI on day 1), SCI-Day 3 group (SCI on day 3), and SCI-Day 7 group (SCI on day 7); these mice were not subjected to any drug treatment. Other C57BL/6 mice were divided into Control+vehicle1 group, Control + MnTBAP group, SCI + vehicle1 group, SCI + MnTBAP group. SCI+DMSO group, SCI + Compound C group, SCI + vehicle2 group, SCI + AAV-scramble control group, and SCI+AAV-TFE3 shRNA group. Mice from Control + MnTBAP and SCI + MnTBAP groups received daily intraperitoneal injections of MnTBAP (dissolved into normal saline) starting from 3 days before SCI until they were euthanized; mice in Control+vehicle1 and SCI + vehicle1 groups were provided the same dose and course of normal saline. For mice in SCI + Compound C group, daily intraperitoneal injection of Compound C (dissolved into DMSO) was performed 7 days before operation, and then continued to receive the drug until the mice were euthanized; in the SCI + DMSO group, the mice received equal doses of DMSO at the same time points. 28 days before the operation, mice in SCI + scramble control group and SCI + TFE3 shRNA group received a dose of 2ul viral vectors in PBS, respectively, by *in situ* injections in spinal cord tissue via a microsyringe; while the SCI+vehicle2 with an equal volume of PBS using the same protocol. ATG5^-/+^ mice and ATG5^+/+^ mice were randomly divided into ATG5^-/+^ + Control group, ATG5^+/+^ + Control group, ATG5^-/+^ + SCI group, ATG5^+/+^ + SCI group. TFE3-KI/wt mice and TFE3-wt/wt mice were arranged for TFE3-wt/wt + SCI group TFE3-KI/wt + SCI group. These transgenic mice were not subjected to any drug treatment. Group assignments and associated procedures and treatments are presented in Table S1.

### SCI model and survival rate analysis

SCI contusion was performed in mice as previously described 3. In brief, after anesthesia with 0.3% sodium pentobarbital solution (0.2 ml/10g, ip), mice were placed on a constant heating pad to maintain temperature at 37°C throughout the SCI procedure. A laminectomy was performed at the level of T9-T10 to expose the dorsal cord surface without disrupting the dura. After that, a 10g weight was dropped from 1.5 cm onto the exposed spinal cord to inducing moderate SCI contusion while keeping the dura intact. Then, muscle and skin were sutured in layers with 4-0 silk and needle. After SCI, the mice received manual urinary bladder emptying three times daily until bladder reflexes were established. Mice assigned to the sham procedure received anesthesia and a laminectomy but without SCI. The survival of mice subjected to SCI was evaluated on the following 28 days. Survival was monitored at the time points of days 1, 3, 7, 14, 21, and 28 after SCI.

### Cell culture and groups

PC12 cells were purchased from the Cell Storage Center of Wuhan University (Wuhan, China). Before any treatment, PC12 cells were cultured for 3 days in the presence of β-NGF (50 ng/mL, Catalog No. 11050-HNAC, Sino Biological, Beijing, China) for induction to differentiate into neuron-like cells. The cells were cultured in an incubator at 37℃ with 5% CO_2_ and 95% air. Heat-inactivated 10% fetal bovine serum and 1% penicillin/streptomycin solution were added to RMPI medium 1640 for cell culture. Different concentrations (140, 280 and 560 μM) of TBHP solution was administered to PC12 cells for 1 h to mimic oxidative stress damage experienced by neurons after SCI. Cells were also pre-treated with 5 mM NAC for 2 h to produce an antioxidant effect. According to the treatments, the cells were divided into Control, L-TBHP, M-TBHP, H-TBHP, and H-TBHP+NAC.

### BMS and footprint analysis

BMS scoring and footprint analysis were conducted to detect variations in locomotive function of hind limbs in mice at 0, 1, 3, 7, 14, 21, and 28 days post-injury. BMS scoring of mice after SCI were scored from 0-9 points based on the scoring system (posterior ankle joint mobility, coordination, paw posture, trunk stability, and tail posture). Footprint analysis compared the trajectories of the movements of the hind limbs obtained on a white runway after immersing hind limbs of different groups of mice in the red dye. These two behavioral experiments were performed by two investigators who were blinded to the experimental grouping.

### Tissue slides preparation and HE, Nissl and Masson staining

On days 1, 3, 7, and 14, mice were deeply anesthetized and euthanized by trans-cardiac perfusion with ice-cold 100mM phosphate-buffered saline (PBS, PH 7.4) followed by addition of 4% (w/v) PFA in PBS. The rostral spinal cord segments (1mm in length, 4mm far from epicenter) and the whole segment (10mm in length, epicenter in middle) were separated and post-fixed in PFA overnight. These samples were washed and embedded in paraffin wax. The paraffin sections (5μm) were cut and mounted on gelatin-coated slides. The rostral segments and the whole segments were prepared for transverse and longitudinal sections, respectively. The transverse and longitudinal sections were stained with HE staining kit as previously described 59, 60. Transverse sections were immersed in 1% cresyl violet acetate for Nissl staining according to the manufacturer's instructions. Nissl positive cells were visualized to define the motor neurons in the area of anterior horns, based on previous criteria 61. For Masson staining, longitudinal sections were mordanted in 10% potassium dichromate and 10% trichloroacetic acid, and nuclei were stained with hematoxylin. After that, slides differentiated with hydrochloric acid and ethanol returned to blue with weak ammonia and stained with Masson solution. This staining protocol was performed as previously described 62. Finally, images were photographed using a light microscope (Olympus, Tokyo, Japan).

### Quantitative real-time polymerase chain reaction

Total RNA was extracted from spinal cord lesion after treated with Trizol reagent. According to the manufacturer's instructions of Prime Script Ⅱ 1st Strand cDNA Synthesis Kit (6210B, TAKARA BIO INC), cDNA was synthesized by reverse transcriptase. Then based on data in Genbank, the primer sequences were synthesized by Nanjing Zoonbio Biotechnology Co., Ltd. The primer sequences are listed in Table S2. Real-time PCR was carried out by using SYBR Premix Ex Taq (RR420A, TAKARA BIO INC). The conditions of reaction were as follows: denaturation at 95°C for 30 s, annealing at 65°C for 30 s, extension at 72°C for 45 s for 30 cycles, and the signal was detected at 72°C. Finally, these targeted mRNAs were normalized to *β-actin* mRNA.

### Western blot analysis

Spinal cord segments (1.0 cm, in length) at the injury epicenter and cell samples were dissected and immediately stored at -80°C for future Western blot analysis as previously published 63. A part of the samples was processed by extracting proteins with a lysis buffer. Other samples were processed by extracting cytoplasmic protein and nuclear protein with NE-PER™ Nuclear and Cytoplasmic Extraction Reagents. Subsequently, protein concentration was determined by the BCA test kit. 65μg protein was separated on 12% sodium dodecyl sulfate-polyacrylamide gel electrophoresis (SDS-PAGE) and transferred onto a polyvinylidene fluoride membrane. Blocked with 10% non-fat milk, the membranes incubated with primary antibodies at 4°C overnight. Then, the membrane was incubated with HRP-conjugated IgG second antibody at room temperature for 1h. Information regarding diluted concentrations of antibodies is indicated in Table S3. Bands signals were visualized and analyzed by a ChemiDicTM XRS + Imaging System (Bio-Rad) via ECL immune-detection kit.

### Immunofluorescence

Transverse sections of spinal cord segments and PC12 cell slides were prepared for immunofluorescence as previously published 64. Samples were restored by high-pressure antigen retrieval after dewaxing and hydration. Next, the sections were blocked with 5% bovine serum albumin in phosphate-buffered saline (PBS) for 30 min at 37°C and then were immune-stained with primary antibodies overnight at 4°C. On the following day, the sections were re-incubated with secondary antibodies, goat anti-rabbit IgG H&L (Alexa Fluor® 488), and goat anti-mouse IgG H&L (Alexa Fluor® 647) for 1h at 37°C, and counterstained with DAPI staining solution. The information of diluted concentration of antibodies is listed in Table S3. Images were captured using Nikon ECLIPSE 80i microscope under the same laser intensity by an investigator who was blinded to experimental grouping.

### TUNEL staining

As described previously, TUNEL co-staining with NeuN in spinal cord sections was performed on day1 or day 3 after SCI. After permeabilization with 0.1% PBS-Triton X-100 for 15 min, spinal cord slides were blocked in 10% bovine serum albumin for 1 h. Then sections were incubated with a primary antibody against NeuN (1:100) at 4°C overnight. After washing with PBS, the slides were incubated with 0.1% goat anti-mouse IgG H&L (Alexa Fluor® 647) in TUNEL staining liquid for 1h at room temperature. Images were observed by a Nikon ECLIPSE 80i microscope (Tokyo, Japan).

### GFP-mCherry-LC3 assay

Ad-mCherry-GFP-LC3B is an adenovirus that expresses the mCherry-GFP-LC3B fusion protein, which can be used to detect autophagy flux after infecting cells. 8×10^3^ cells seeded in 12-well plates were infected with virus diluted in a fresh medium under a multiplicity of infection (MOI) values of 30. After 48 h of infection, cells were observed under a Nikon ECLIPSE 80i microscope (Tokyo, Japan). The relative intensity of fluorescence was analyzed with Image J software. GFP degrades in an acidic environment while RFP does not. Therefore, yellow spots (formed out of the overlap between red and green) indicate autophagosomes, while, red spots indicate autolysosomes. If autophagy flux is increased, the red signal will dominate over yellow. If autophagy flux is suppressed, there will be more yellow signal than red signal.

### LC3 process assay in spinal cord slides

On day1 or day 3 after SCI, LC3 process assay in spinal cord sections was performed as previously described 65. After animals were euthanized, the spinal cord was collected in oxygenated artificial cerebral spinal fluid. 400mm spinal cord sides were cut by using a Leica 1000 Plus vibratome. Then, the sections were incubated in the oxygenated artificial cerebral spinal fluid with or without 100 mM chloroquine for 2h at room temperature. After that, the sections were homogenized in RIPA buffer containing protease and phosphatase inhibitors and centrifuged at 20,000 g at 4°C for 20 min to collect protein. Finally, the protein extractions were analyzed by Western blot.

### DCFH-DA staining

PC12 cells were seeded at 5×10^4^ per well in 12 well plates containing rounded cell culture slides that had been sterilized and coated with a poly-L-lysine solution. When the cell density reaches 50%, the cells received their assigned drug treatments. After the end of the cell treatment, the drug-containing medium was removed, and then the probe diluted to 10 μM with the serum-free medium was added at 500 μL per well and incubated at 37°C. After 20 min, wells were gently cleaned with serum-free medium to remove DCFH-DA that did not enter the cells. The round cell culture slides were then removed from the wells and the slides were mounted on clean slides using DAPI. Images were captured by a Nikon ECLIPSE 80i microscope (Tokyo, Japan).

### Cell Counting Kit 8

PC12 cells were seeded in 96-well plates at 1×10^4^ cells per well. After the cell density of each well reached 50%, the cells were treated with drugs. The drug-containing medium was discarded, followed by gently adding 100 μL of fresh cell culture medium to each well after washing three times with PBS solution. Subsequently, the CCK-8 solution was added at 10 μL per well and then incubated at 37°C for 3 h. Absorbance of each well was measured at 450 nm in a microplate reader.

### Enzyme-linked immunosorbent assay

Spinal cord tissues were homogenized in PBS and then frozen and thawed repeatedly in liquid nitrogen. After that, the homogenate was centrifuged at 10,000 g for 10 minutes at 4°C, and its supernatant was collected for ELISA. The levels of 8-OHdG, AOPP, and MDA in spinal cord lesions were detected by using ELISA kits according to the manufacturer's protocols (Boyun Biotech, Shanghai, China). Finally, the quantifications of 8-OHdG, AOPP, and MDA were determined by a microplate reader at 550 nm with correction wavelength at 450 nm.

### Immunoprecipitation

The protocol of immunoprecipitation was performed as previously described 66. Spinal cord tissues were homogenized in IGEPAL CA-630 buffer (50 mM Tris-HCl, 1% IGEPAL CA-630, 10 mM EDTA, 150 mM NaCl, 50 mM NaF, 1 μM leupeptin and 0.1 μM aprotinin). After centrifugation, the supernatant of homogenate was collected for immunoprecipitation. Based on the manufacturer's instructions of the Pierce Crosslink immunoprecipitation kit, the primary antibody was covalently immobilized on protein A/G agarose. Samples were incubated with immobilized antibody beads at 4°C for 2 h. Samples were also subjected to immunoprecipitation with IgG isotype control. After the immunoprecipitation and washing with TBS liquid, samples eluted with glycine-HCl (0.1 M, pH 3.5), and the immunoprecipitants were subjected to Western blotting using related primary antibodies.

### Statistical analysis

Statistical analyses were carried out by using SPSS software version 19 (Chicago, IL, USA). All data were presented as mean ± Standard Error of Mean (SEM). One-way Analysis of Variance (ANOVA) with LSD (equal variances assumed) post hoc analysis or Dunnett's T3 (equal variances not assumed) method were used for the evaluation of significant differences between two groups in three or four groups, excepting the survival rate. Independent-sample t-tests were used for significant difference test in two groups. The Kaplan-Meier-rank test was performed to determine the differences in survival between groups. P-values less than 0.05 were considered statistically significant.

## Supplementary Material

Supplementary figures and tables.

## Abbreviations

SCI: spinal cord injury
UPR: unfolded protein response
ER stress: endoplasmic reticulum stress
ROS: reactive oxygen species
bHLH-LZ: basic helix-loop-helix leucine zipper
MITF: microphthalmia-associated transcription factor
TFEB: transcription factor EB
TFE3: transcription factor E3
TFEC: transcription factor EC
CLEAR: coordinated lysosomal expression and regulation
ATG5: autophagy-related gene 5
MAP1LC3/LC3: microtubule-associated protein 1 light chain 3
CTSD: cathepsin d
LAMP2: lysosomal-associated membrane protein 2
UB: ubiquitin
SQSTM1: sequestosome 1
PGC1α: peroxisome proliferator-activated receptor-γ coactivator 1α
GPX 3: glutathione peroxidase 3
SOD 1: superoxide dismutase 1
HO1: heme oxygenase 1
TRX: thioredoxin
AMPK: adenosine 5'-monophosphate-activated protein kinase
mTOR: mammalian target of rapamycin
FOXO3a: forkhead box o3a
SKP2: s phase kinase-associated protein 2
CARM1: coactivator-associated arginine methyltransferase 1
CQ: chloroquine
GRP78: glucose-regulated protein 78
PDI: protein disulfide isomerase
PERK: protein kinase-like endoplasmic reticulum kinase
eIF2α: eukaryotic initiation factor 2α
ATF4: activating transcription factor 4
CHOP: C/EBP-homologous protein
CASP12: caspase 12
CASP3: caspase 3
TUNEL: TdT-mediated dUTP nick end labeling
HE staining: hematoxylin-eosin staining
MAP2: microtubule-associated protein 2
8-OHdG: 8-hdroxydeoxyguanosine
AOPP: advanced oxidation protein products
MDA: malondialdehyde
BMS: Basso mouse scale
MnTBAP: Mn (ⅡI) tetrakis (4-benzoic acid) porphyrin
TBHP: tert-butyl hydroperoxide
NAC: N-Acetyl-L-cysteine
AAV: adeno-associated Virus
shRNA: short hairpin RNA
KI/wt: knock in/wild type
DMSO: dimethyl sulfoxide
Baf-A1: bafilomycin-A1
WB: Western blotting
qPCR: quantitative real-time polymerase chain reaction
IP: immunoprecipitation
CCK 8: cell counting kit 8
ELISA: enzyme-linked immunosorbent assay
CNS: central nervous system
