# Supplementary material for: TFE3, a potential therapeutic target for Spinal Cord Injury via augmenting autophagy flux and alleviating ER stress
Source: Theranostics. 2020 Jul 23;10(20):9280–302. doi: 10.7150/thno.46566 (PMC7415792; doi:10.7150/thno.46566)
Supplement: Supplementary file 1 — Supplementary figures and tables. [file thnov10p9280s1.pdf]

Supplementary Information

for

TFE3, a potential therapeutic target for spinal cord injury via ameliorating autophagy flux disruption aggravated ER stress

Kailiang Zhou et al.

Figures

Figure S1

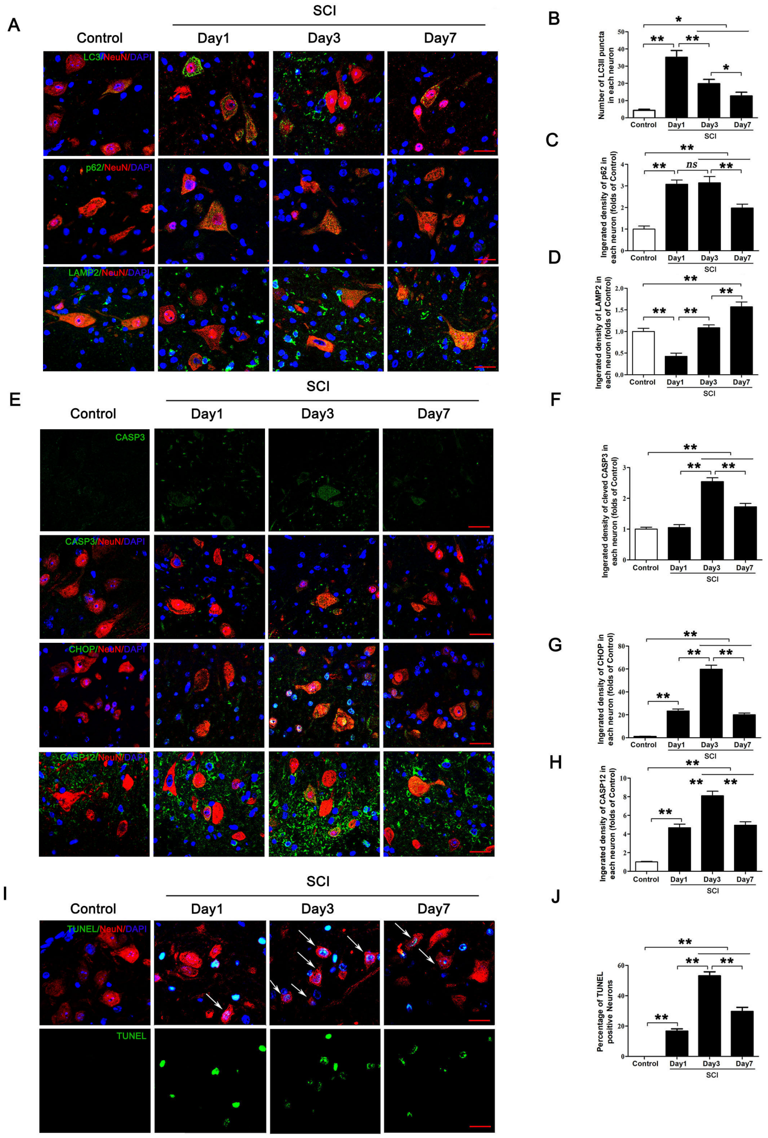

Figure S2

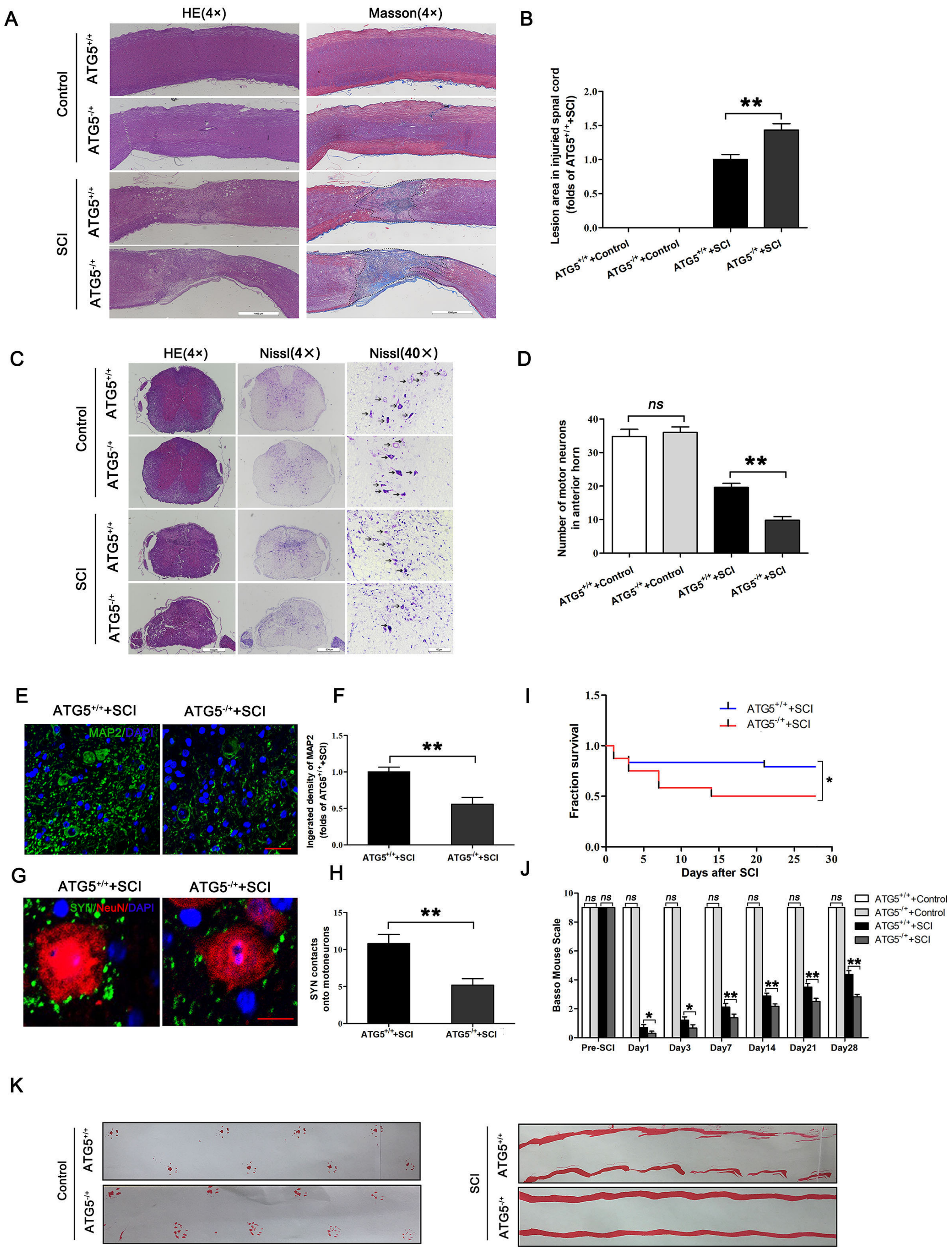

A
HE(4×)
Masson(4×)
Control
SCI
ATG5+/+
ATG5-/+
B
Lesion area in injuried spinal cord (folds of ATG5+/+ +SCI)
**
ATG5+/+ +Control
ATG5-/+ +Control
ATG5+/+ +SCI
ATG5-/+ +SCI
C
HE(4×)
Nissl(4×)
Nissl(40×)
D
ns
**
Number of motor neurons in anterior horn
E
ATG5+/++SCI
ATG5-/++SCI
MAP2/DAPI
F
Ingerated density of MAP2 (folds of ATG5+/++SCI)
G
SYN/NeuN/DAPI
H
SYN contacts onto motoneurons
I
ATG5+/++SCI
ATG5-/++SCI
Fraction survival
Days after SCI
*
J
Basso Mouse Scale
ATG5+/++Control
ATG5-/++Control
ATG5+/++SCI
ATG5-/++SCI
Pre-SCI
Day1
Day3
Day7
Day14
Day21
Day28
ns
K
Control
SCI
ATG5+/+
ATG5-/+

Figure S3

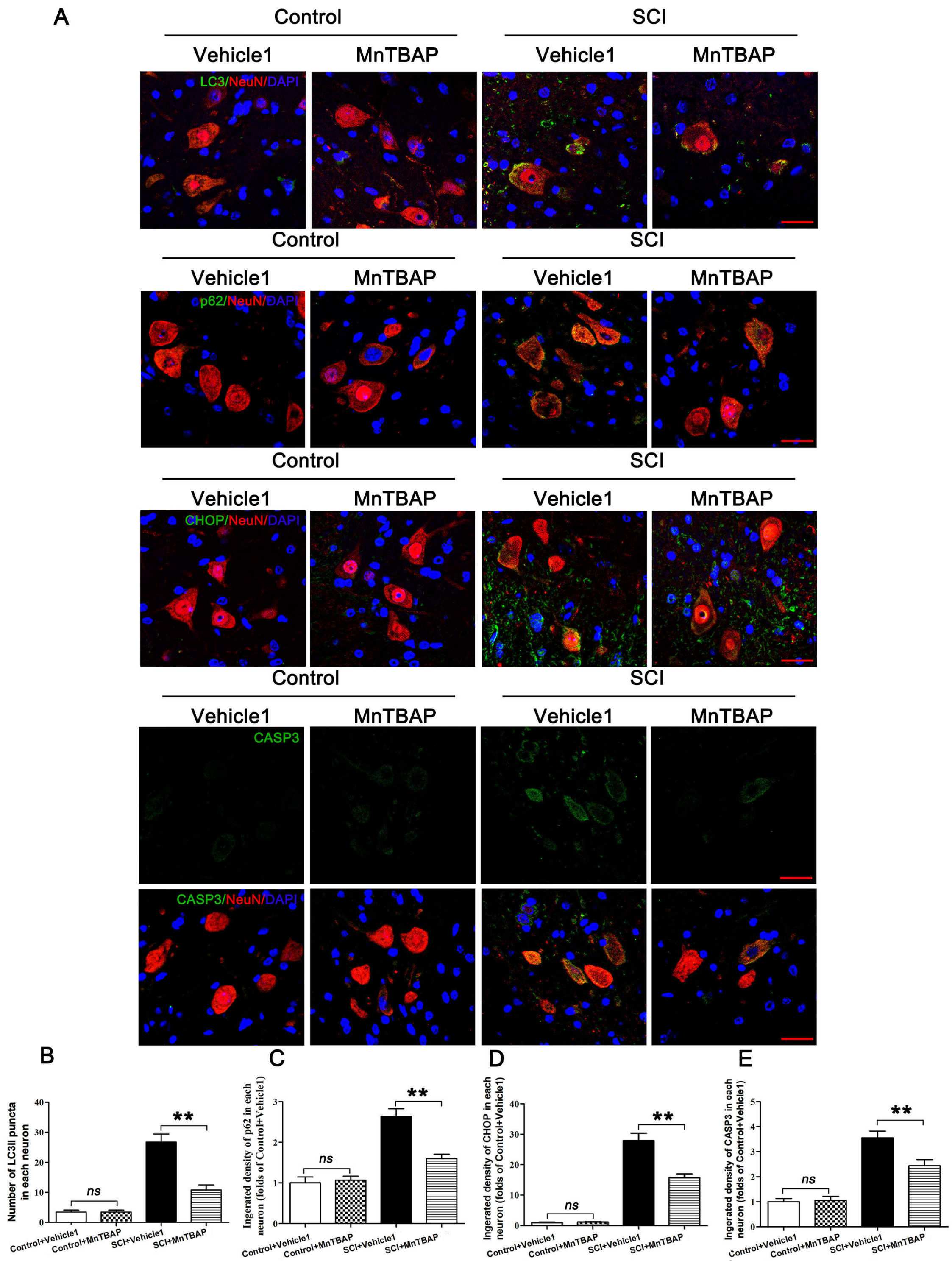
A
Control
SCI
Vehicle1
MnTBAP
LC3/NeuN/DAPI
p62/NeuN/DAPI
CHOP/NeuN/DAPI
CASP3
CASP3/NeuN/DAPI
B
C
D
E
Number of LC3II puncta in each neuron
Ingerated density of p62 in each neuron (folds of Control+Vehicle1)
Ingerated density of CHOP in each neuron (folds of Control+Vehicle1)
Ingerated density of CASP3 in each neuron (folds of Control+Vehicle1)
ns
**
Control+Vehicle1
Control+MnTBAP
SCI+Vehicle1
SCI+MnTBAP

Figure S4

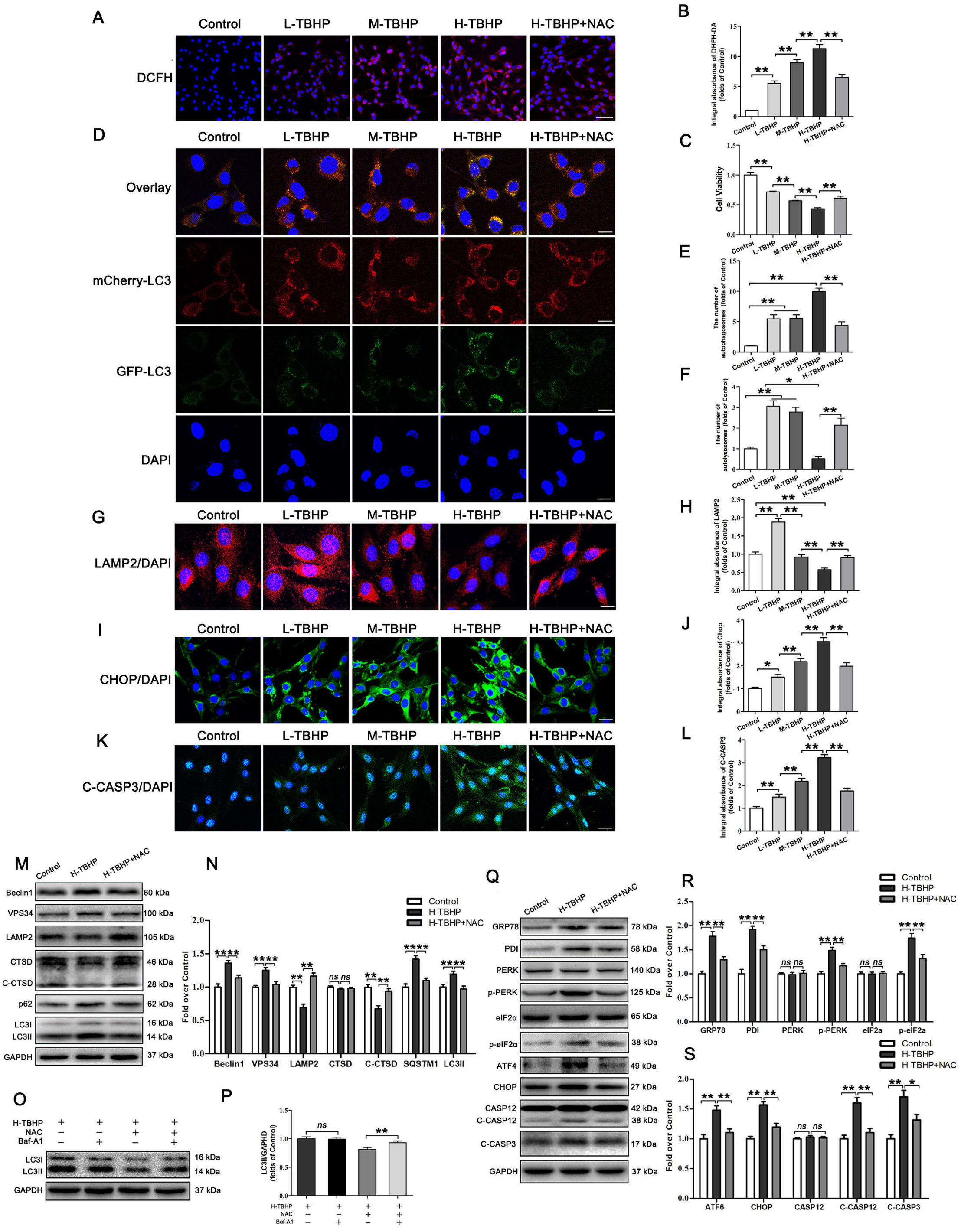
A
Control
L-TBHP
M-TBHP
H-TBHP
H-TBHP+NAC
DCFH
B
Integral absorbance of DHFH-DA (folds of Control)
C
Cell Viability
D
Overlay
mCherry-LC3
GFP-LC3
DAPI
E
The number of autophagosomes (folds of Control)
F
The number of autolysosomes (folds of Control)
G
LAMP2/DAPI
H
Integral absorbance of LAMP2 (folds of Control)
I
CHOP/DAPI
J
Integral absorbance of Chop (folds of Control)
K
C-CASP3/DAPI
L
Integral absorbance of C-CASP3 (folds of Control)
M
Beclin1
VPS34
LAMP2
CTSD
C-CTSD
p62
LC3I
LC3II
GAPDH
60 kDa
100 kDa
105 kDa
46 kDa
28 kDa
62 kDa
16 kDa
14 kDa
37 kDa
N
Fold over Control
Beclin1
VPS34
LAMP2
CTSD
C-CTSD
SQSTM1
LC3II
O
H-TBHP
NAC
Baf-A1
P
LC3II/GAPHD (folds of Control)
Q
GRP78
PDI
PERK
p-PERK
eIF2α
p-eIF2α
ATF4
CHOP
CASP12
C-CASP12
C-CASP3
78 kDa
58 kDa
140 kDa
125 kDa
65 kDa
38 kDa
49 kDa
27 kDa
42 kDa
17 kDa
R
GRP78
PDI
PERK
p-PERK
eIF2a
p-eIF2a
S
ATF6
CHOP
CASP12
C-CASP12
C-CASP3

Figure S5

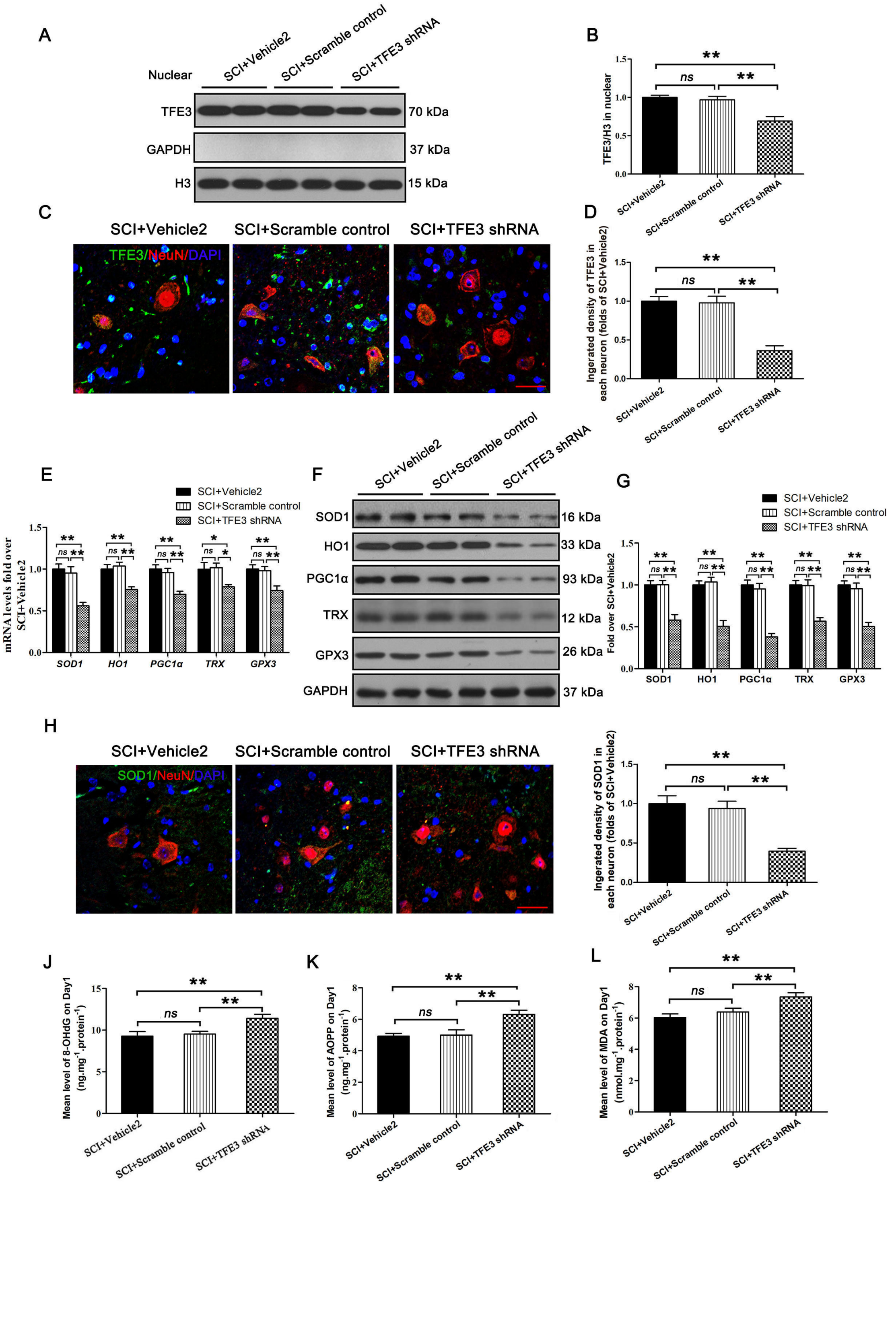
A
Nuclear
SCI+Vehicle2
SCI+Scramble control
SCI+TFE3 shRNA
TFE3
70 kDa
GAPDH
37 kDa
H3
15 kDa
B
TFE3/H3 in nuclear
C
SCI+Vehicle2
SCI+Scramble control
SCI+TFE3 shRNA
TFE3/NeuN/DAPI
D
Ingerated density of TFE3 in each neuron (folds of SCI+Vehicle2)
E
mRNA levels fold over SCI+Vehicle2
SOD1
HO1
PGC1α
TRX
GPX3
F
SOD1
16 kDa
HO1
33 kDa
PGC1α
93 kDa
TRX
12 kDa
GPX3
26 kDa
GAPDH
37 kDa
G
Fold over SCI+Vehicle2
H
SOD1/NeuN/DAPI
Ingerated density of SOD1 in each neuron (folds of SCI+Vehicle2)
J
Mean level of 8-OHdG on Day1 (ng.mg^{-1}.protein^{-1})
K
Mean level of AOPP on Day1 (ng.mg^{-1}.protein^{-1})
L
Mean level of MDA on Day1 (nmol.mg^{-1}.protein^{-1})

Figure S6

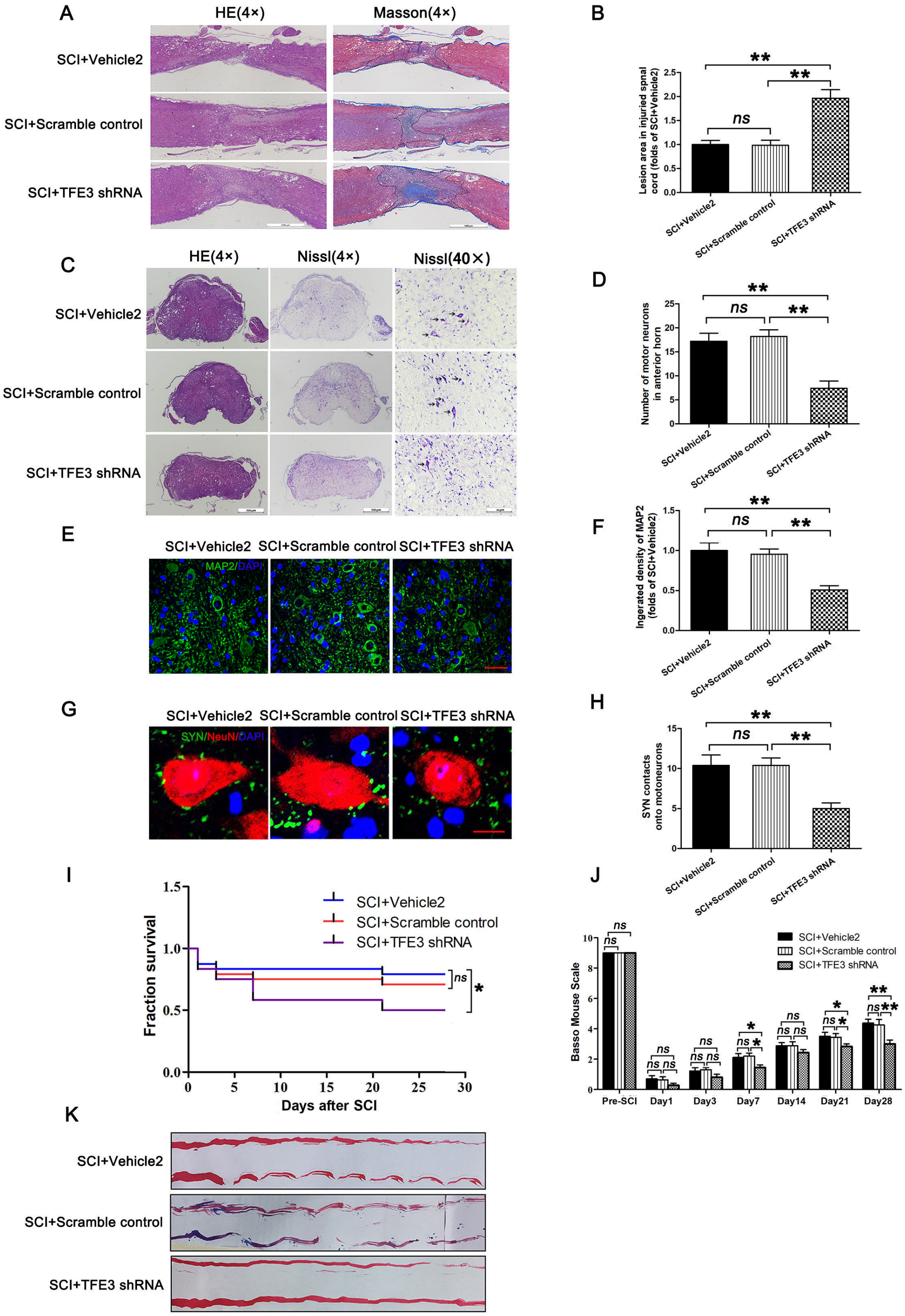
A
HE(4×)
Masson(4×)
SCI+Vehicle2
SCI+Scramble control
SCI+TFE3 shRNA
B
Lesion area in injuried spinal cord (folds of SCI+Vehicle2)
C
HE(4×)
Nissl(4×)
Nissl(40×)
D
Number of motor neurons in anterior horn
E
MAP2/DAPI
F
Ingerated density of MAP2 (folds of SCI+Vehicle2)
G
SYN/NeuN/DAPI
H
SYN contacts onto motoneurons
I
Fraction survival
Days after SCI
J
Basso Mouse Scale
Pre-SCI
Day1
Day3
Day7
Day14
Day21
Day28
K
ns
**
*

Figure S7

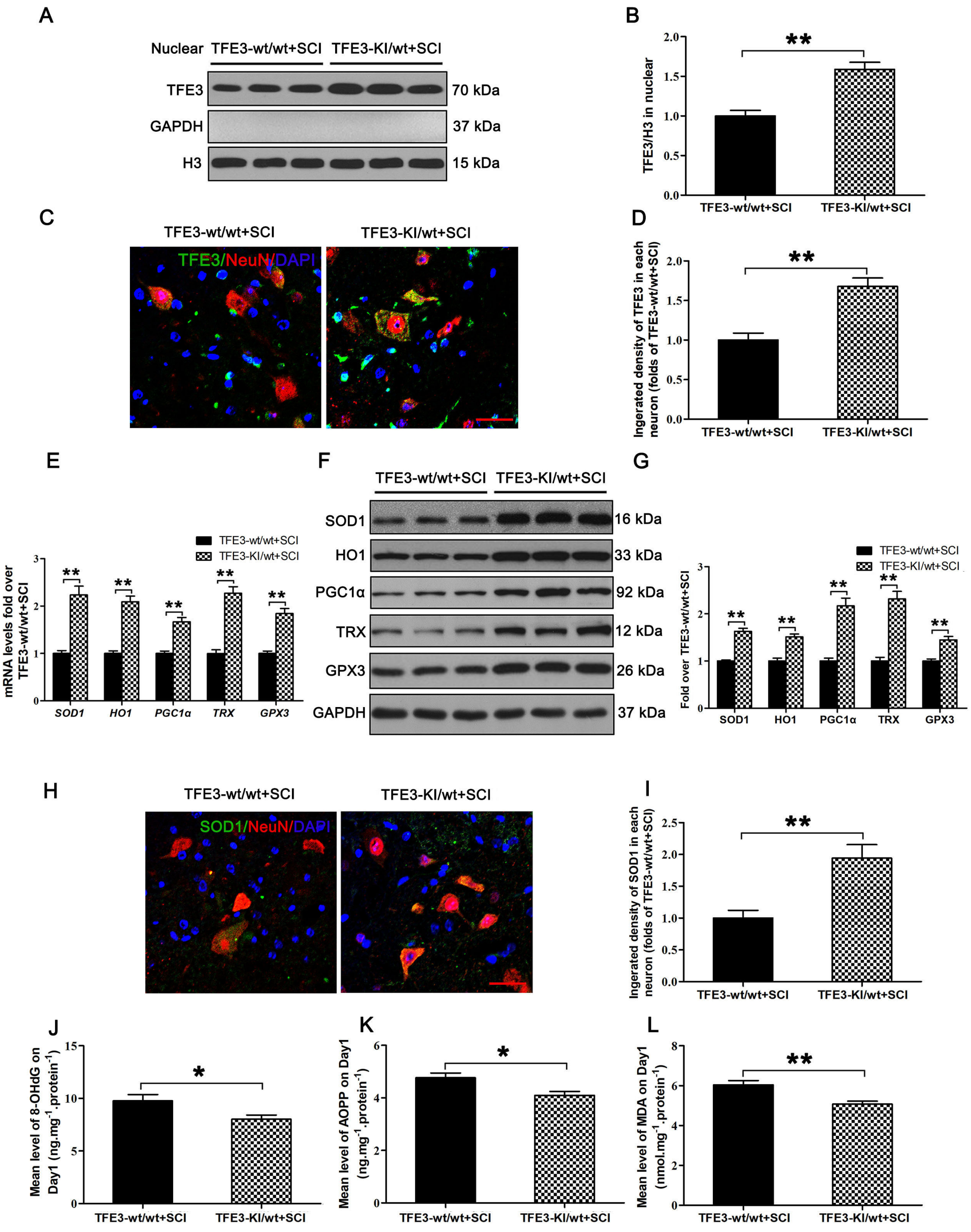
A
Nuclear TFE3-wt/wt+SCI TFE3-KI/wt+SCI
TFE3 70 kDa
GAPDH 37 kDa
H3 15 kDa
B
TFE3/H3 in nuclear
TFE3-wt/wt+SCI TFE3-KI/wt+SCI
C
TFE3-wt/wt+SCI TFE3-KI/wt+SCI
TFE3/NeuN/DAPI
D
Ingerated density of TFE3 in each neuron (folds of TFE3-wt/wt+SCI)
E
mRNA levels fold over TFE3-wt/wt+SCI
SOD1 HO1 PGC1α TRX GPX3
F
SOD1 16 kDa
HO1 33 kDa
PGC1α 92 kDa
TRX 12 kDa
GPX3 26 kDa
GAPDH 37 kDa
G
Fold over TFE3-wt/wt+SCI
H
SOD1/NeuN/DAPI
I
Ingerated density of SOD1 in each neuron (folds of TFE3-wt/wt+SCI)
J
Mean level of 8-OHdG on Day1 (ng.mg-1.protein-1)
K
Mean level of AOPP on Day1 (ng.mg-1.protein-1)
L
Mean level of MDA on Day1 (nmol.mg-1.protein-1)

Figure S8

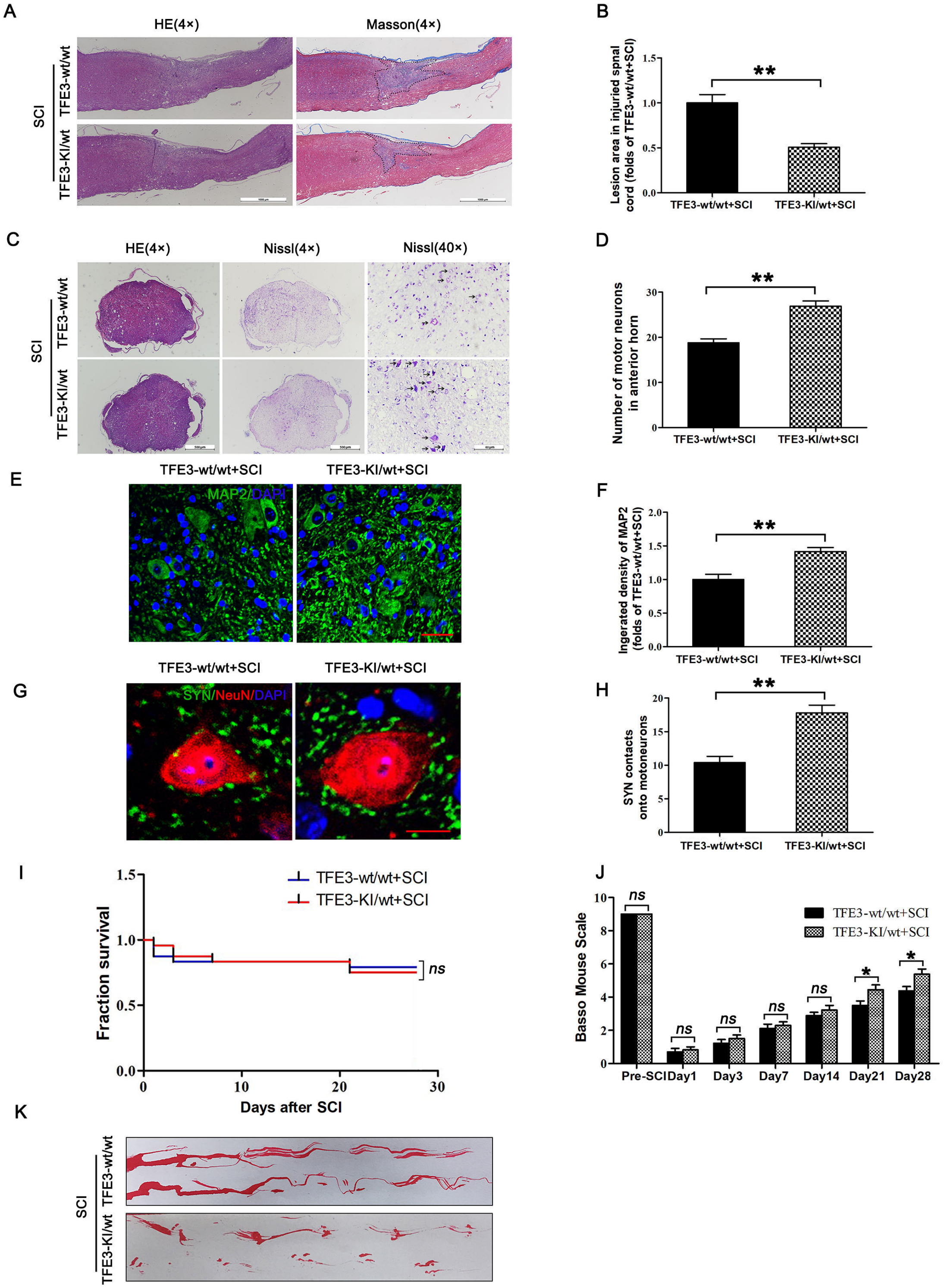
A
HE(4×)
Masson(4×)
SCI
TFE3-wt/wt
TFE3-KI/wt
B
Lesion area in injuried spinal cord (folds of TFE3-wt/wt+SCI)
TFE3-wt/wt+SCI
TFE3-KI/wt+SCI
C
HE(4×)
Nissl(4×)
Nissl(40×)
D
Number of motor neurons in anterior horn
E
TFE3-wt/wt+SCI
TFE3-KI/wt+SCI
MAP2/DAPI
F
Ingerated density of MAP2 (folds of TFE3-wt/wt+SCI)
G
SYN/NeuN/DAPI
H
SYN contacts onto motoneurons
I
Fraction survival
Days after SCI
ns
J
Basso Mouse Scale
Pre-SCI
Day1
Day3
Day7
Day14
Day21
Day28
K

Figure legends

Figure S1. SCI leads to autophagy flux blockade and ER stress-induced apoptosis in motor neurons. （A）Images (30×) of spinal cord sections from the indicated groups at Day 3 stained with antibodies against LC3/NeuN, p62/NeuN, and LAMP2/NeuN; scale bar: 25 μm. (B-D) Quantification of immunofluorescence from (A) indicating the mean number of LC3II and optical density of p62 and LAMP2 in motor neurons at the spinal cord. (E) Images (30×) of spinal cord sections stained with antibodies against CASP3/NeuN, CHOP/NeuN, and CASP12/NeuN; scale bar: 25 μm. (F-H) Quantification data from (E) showing the optical density of CASP3, CHOP, and CASP12 in motor neurons. (I, J) Immunofluorescence image (30×) and quantification of cells single positive for TUNEL staining in the indicated groups; write arrows indicating the TUNEL-positive Neurons; scale bar: 25 μm. n=6, *ns* stands for not significant, **P*<0.05, ***P*<0.01.

Figure S2. Inhibition of autophagy activity depresses functional recovery after SCI. (A) Longitudinal spinal cord sections from the indicated groups at Day 14 were analyzed by HE staining and Masson staining; scale bar: 1000 μm. (B) Quantitative analysis of Masson positive lesions in the spinal cords of each group. (C) Transverse spinal cord sections from each group were analyzed at Day 14 by HE staining and Nissl staining; black arrows indicating the motoneurons; scale bar: 500 μm and 125 μm. (D) Quantitative analysis of Nissl positive motor neurons in the anterior horn of spinal cord from each group. (E) Images (30×) of the spinal cord sections in each group stained with antibodies against MAP2; scale bar: 25 μm. (F) Quantification data from (E) showing the optical density of MAP2 in the injured spinal cord at Day 28. (G) Images (150×) of spinal cord sections below the injury (T11-T12) stained at Day 28 with antibodies against SYN/NeuN; scale bar: 5 μm. (H) Corresponding quantification of the number of synapses contacting motor neurons. (I) The analysis of survival rate of $ATG5^{-/+}$ mice and

$ATG5^{+/+}$ mice subjected to SCI at Day1, Day3, Day7, Day14, Day21, and Day28. (J) Basso mouse scale (BMS) for the indicated groups and time points (n= 6-12). (K) Photos of mice footprints at Day 28 after SCI. n=6 (except for BMS), *ns* stands for not significant, **P*<0.05, ***P*<0.01.

Figure S3. ROS-induced lysosomal dysfunction results in autophagy flux blockade and ER stress-induced apoptosis in motor neurons following SCI. (A) Images (30×) of spinal cord sections from the indicated groups stained with antibodies against LC3/NeuN, p62/NeuN at Day1, and stained with antibodies against CHOP/NeuN and CASP3/NeuN at Day3; scale bar: 25 μm. (B-D) Corresponding quantification of the mean number of LC3II and optical density of p62, CHOP and CASP3 in motor neurons at the spinal cord. n=6, *ns* stands for not significant, **P*<0.05, ***P*<0.01.

Figure S4. Lysosomal dysfunction due to ROS leads to autophagy flux blockade and ER stress-induced apoptosis in PC12 cells. PC12 cells were treated with TBHP in low (140 μM, L-TBHP), medium (280 μM, M-TBHP) or high (560 μM, H-TBHP) concentrations, and the control treatment, for 1h. For oxidation depression, PC12 cells were pretreated with ROS scavenger, NAC (5 mM), for 1h then were incubated in culture medium with H-TBHP for 1h. (A) PC12 cells of the indicated groups were stained with DCFH-DA; scale bar: 50 μm. (B) Quantification of fluorescence data from (A) showing the optical density of DCFH in the cells. (C) CCK8 indicating the cell viability of these groups. (D) GFP-mCherry-LC3 staining for the indicated groups; scale bar: 10 μm. (E, F) Corresponding quantification of the optical density of GFP-LC3 and GFP-mCherry-LC3 in PC12 cells. (G, H) Immunofluorescence staining and its quantification of LAMP2 expression in each group; scale bar: 10 μm. (I-L) Immunofluorescence staining and corresponding quantification of CHOP and LAMP2 expression in the indicated groups; scale bar: 20 μm. (M) Western blot analysis of autophagy flux markers in the Control,

H-TBHP and H-TBHP+NAC groups. (N) Densitometric analysis of band data from (M) normalized to loading control GAPDH. (O) Western blotting of LC3II in the cells of H-TBHP and H-TBHP+NAC groups cultured in the presence or absence of Baf-A1. (P) Densitometric analysis of LC3II data from (O) normalized to loading control GAPDH. (Q) Western blot analysis of ER stress-induced apoptosis markers in the indicated groups. (R, S) Quantitative analyses of data from (Q) normalized to loading control GAPDH. n=5, *ns* stands for not significant, **P*<0.05, ***P*<0.01.

Figure S5. Underexpression of TFE3 reduces the expression of antioxidase and decreases ROS elimination after SCI. (A) Western blot analysis of TFE3 from the nuclear extract protein as indicated in the spinal cord lesion 3 days after SCI from SCI +Vehicle2 mice and mice injected with AAV-Scramble or AAV-shTFE3. (B) Densitometric analysis of (A) TFE3 normalized to loading control GAPDH. (C, D) Immunofluorescence image (30×) and its quantification of TFE3 expression in motor neurons in the indicated groups; scale bar: 25 µm. (E) Relative mRNA level of anti-oxidation markers, *Sod1, Ho1, Pgc1α, Trx, and Gpx3* in the lesion normalized to control β-actin at Day3. (F) Western blotting of the anti-oxidation markers in the injured spinal cord of each group. (G) Densitometric analysis of the data from (F) normalized to loading control GAPDH. (H, I) Immunofluorescence image (30×) and corresponding quantification of SOD1 expression in motor neurons in the indicated groups; scale bar: 25 µm. (J-L) ELISA of 8-OHdG, AOPP, and MDA in spinal cord lesions. n=6, *ns* stands for not significant, **P*<0.05, ***P*<0.01.

Figure S6. Underexpression of TFE3 suppresses functional recovery after SCI. (A) The longitudinal sections of spinal cords from the indicated groups were analyzed by HE staining and Masson staining at Day14; scale bar: 1000 μm. (B) Quantitative analysis of Masson positive lesions in the spinal cord of each group. (C) The transverse sections of

spinal cords from each group were analyzed by HE staining and Nissl staining at Day 14; black arrows indicating the motoneurons; scale bar: 500 μ m and 125 μ m. (D) Quantitative analysis of Nissl positive neurons in the anterior horn of the spinal cord. (E, F) Images (30×) and its quantification of MAP2 expression in neurons at the anterior horn at Day 14; scale bar: 25 μ m. (G) Images (150×) of spinal cord sections below the injury (T11-T12) in three groups stained with antibodies against SYN/NeuN at Day14; scale bar: 5 μ m. (H) Corresponding quantification of the number of synapses contacting motor neurons. (I) The analysis of survival rate of indicated groups at Day1, Day3, Day7, Day14, Day21, and Day28 after SCI. (J) BMS results at indicated groups and time points (n=6-12). (K) Photos of mice footprints in three groups on Day28 after SCI. n=6 (except for BMS), *ns* stands for not significant, *$P<0.05$, **$P<0.01$.

Figure S7. Overexpression of TFE3 promotes the expressions of antioxidase and the elimination of ROS after SCI. (A, B) Western blot analysis and corresponding quantification of TFE3 from the nuclear extract protein of spinal cord lesions from TFE3-KI/wt mice and TFE3-wt/wt mice subjected with SCI at Day3. (C) Image (30×) of spinal cord sections from the indicated groups at Day3 stained with antibodies against SOD1/NeuN,; scale bar: 25 μ m. (D) Quantification of immunofluorescence from (C) showing the optical density of SOD1 in motor neurons at the spinal cord. (E) Relative mRNA levels of anti-oxidation markers, *Sod1, Ho1, Pgc1 α, Trx, and Gpx3* in the lesion of the both groups normalized to control *β-actin* at Day3. (F-G) Western blotting and its densitometric analysis of the anti-oxidation markers in the lesion of the both groups normalized to loading control GAPDH. (H, I) Immunofluorescence image (30×) and corresponding quantification of SOD1 expression in motor neurons in the both groups. (J-L) ELISA of 8-OHdG, AOPP, and MDA in the spinal cord lesion from indicated groups at Day 3. n=6, *$P<0.05$, **$P<0.01$.

Figure S8. Overexpression of TFE3 promotes functional recovery after SCI. (A) Longitudinal spinal cord sections from the indicated groups were analyzed by HE staining and Masson staining; scale bar: 1000 μ m. (B) Quantitative analysis of Masson positive lesions in the spinal cord of both groups. (C) The transverse sections of spinal cords from each group were analyzed by HE staining and Nissl staining; black arrows indicating the motoneurons; scale bar: 500 μ m and 125 μ m. (D) Quantitative analysis of Nissl positive motor neurons in the anterior horn of the spinal cord. (E, F) Images (30×) and its quantification MAP2 expression in the anterior horn of the spinal cords from both groups; scale bar: 25 μ m. (G) Images (150×) of spinal cord sections below the injury (T11-T12) in both groups stained with antibodies against SYN/NeuN; scale bar: 5 μ m. (H) Corresponding quantification of the number of synapses contracting motor neurons. (I) The analysis of survival rate of indicated groups at Day1, Day3, Day7, Day14, Day21, and Day28 after SCI. (J) BMS results for indicated groups and time points (n=8-12). (K) Photos of mice footprints in both groups at Day28 after SCI. n=6 (except for BMS), *ns* stands for not significant, **P*<0.05, ***P*<0.01.

Tables

Groups	Mice	Procedure	Treatments	Number
Control	C57BL/6	Sham	/	42
SCI-Day1	C57BL/6	SCI	/	36
SCI-Day3	C57BL/6	SCI	/	36
SCI-Day7	C57BL/6	SCI	/	36
Control+vehicle1	C57BL/6	Sham	Normal saline, *ip*	18
Control + MnTBAP	C57BL/6	Sham	MnTBAP (15 mg/kg), *ip*	18
SCI + vehicle1 group	C57BL/6	SCI	Normal saline, *ip*	24
SCI + MnTBAP	C57BL/6	SCI	MnTBAP (15 mg/kg), *ip*	24
SCI+DMSO	C57BL/6	SCI	DMSO, *ip*	12
SCI + Compound C	C57BL/6	SCI	Compound C (15 mg/kg), *ip*	12
SCI + vehicle2	C57BL/6	SCI	PBS (2ul), *in situ* injection	48
SCI + AAV-scramble control	C57BL/6	SCI	AAV-scramble control (5×10^9 genomic particles, 2ul), *in situ* injection	42

SCI+AAV-TFE3 shRNA	C57BL/6	SCI	AAV-TFE3 shRNA (5×10^9 genomic particles, 2ul), *in situ* injection	48
ATG5$^{-/+}$ + Control	ATG5$^{-/+}$	Sham	/	24
ATG5$^{+/+}$ + Control	ATG5$^{-/+}$	Sham	/	24
ATG5$^{-/+}$ + SCI	ATG5$^{-/+}$	SCI	/	30
ATG5$^{+/+}$ + SCI	ATG5$^{-/+}$	SCI	/	30
TFE3-wt/wt + SCI	TFE3-wt/wt	SCI	/	48
TFE3-KI/wt + SCI	TFE3-KI/wt	SCI	/	48

Table S1. Groups, animals, and treatments in the SCI study.

Primer name	Primer sequences
Atg5	*5'-AGTCAAGTTCAGTGGAGGCAACAG-3'* (forward)<br>*5'-GTGTCTCAGCGAAGCAGTGGTG-3'* (reverse)
Beclin1	*5'-GGACCAGGAGGAAGCTCAGTACC-3'* (forward)<br>*5'-CGCTGTGCCAGATGTGGAAGG-3'* (reverse)
Vps34	*5'-GTCGGTTCCTGTCGCAGAAGTTC-3'* (forward)<br>*5'-TATCCAGGTGCCGGTCTCCAAC-3'* (reverse)
Lamp2	*5'-CCAACTCCAACTCCAACTCCAACC-3'* (forward)<br>*5'-GGCACCTTCTCCTCAGTGATGTTC-3'* (reverse)
Sqstm1/ p62	*5'-ACAACCCGTGTTTCCTTT-3'* (forward)<br>*5'- TGCCACCTTTCACTCACTA-3'* (reverse)
Ctsd	*5'-GGGCATCCAGGTAGTTTT-3'* (forward)<br>*5'-CGTCTTGCTGCTCATTCT-3'* (reverse)
Lc3	*5'-CTACGCCTCCCAAGAAACC-3'*(forward)<br>*5'-AGAGCAACCCGAACATGACT-3'* (reverse)
Mitf	*5'-CATTCTCAAGGCCTCTGTGGACTA-3'* (forward)<br>*5'-GTGCCGAGGTTGTTGGTAAAGGTG-3'* (reverse)
Tfeb	*5'-CAGCAGGTGGTGAAGCAAGAGT-3'*(forward)<br>*5'-TCCAGGTGATGGAACGGAGACT-3'* (reverse)
Tfe3	*5'-CCAGGCTCAGGAACAGGAGA-3'* (forward)<br>*5'-TACTGTTTGACCTGCTGCCG-3'* (reverse)
Sod1	*5'-CGTCGGCTTCTCGTCTTGCTC-3'*(forward)<br>*5'-TCCTGACAACACAACTGGTTCACC-3'* (reverse)
Ho1	*5'-ACCGCCTTCCTGCTCAACATTG-3'* (forward)<br>*5'-CTCTGACGAAGTGACGCCATCTG-3'* (reverse)
Pgc1α	*5'-GTGCCACCGCCAACCAAGAG-3'* (forward)<br>*5'-TTCCTCGTGTCCTCGGCTGAG-3'* (reverse)
Trx	*5'-GCTTGTCGTGGTGGACTTCTCTG-3'* (forward)

	5'-CAGCAACATCCTGGCAGTCATCC-3' (reverse)
Gpx3	*5'-ACACCACCAGCCTCCTTCTTCC-3'* (forward)
	5'-GGCCATCGCGCTCACAGTTG-3' (reverse)
β-actin	*5'-ATGTGGATCAGCAAGCAGGA-3'* (forward)
	5'-AAGGGTGTAAAACGCAGCTCA-3' (reverse)

Table S2. Information of the primer sequences for qPCR.

Antibodies	Concentration for WB	Concentration for IF	Antibodies	Concentration for WB	Concentration for IF
Beclin1	1:1000	/	TFEB	1:500	
ATG5	1:1000	/	TFE3	1:500	1:100
VPS34	1:500	/	SOD1	1:1000	1:100
ATP6VIB2	1:1000	/	HO1	1:1000	/
CTSD	1:500	/	PGC1α	1:1000	/
SQSTM1/p62	1:1000	1:400	TRX	1:1000	/
Ub	1:1000	/	GPX3	1:1000	/
LC3B	1:400	1:100	AMPK	1:500	/
GRP78	1:1000	/	p-AMPK	1:1000	/
PDI	1:1000	/	p-mTOR	1:500	/
PERK	1:1000	/	p-4EBP1	1:800	/
p-PERK	1:1000	/	H3	1:50	/
eIF2α	1:1000	/	p-FOXO3a	1:500	/
p-eIF2α	1:1000	/	SKP2	1:1000	/
ATF4	1:500	/	CARM1	1:500	/
CHOP	1:800	1:100	GAPDH	1:1000	/
LAMP2	1:1000	1:200	NeuN	1:400	/
CASP3	/	1:150	MAP2	1:200	/
CASP12	1:1000	1:500	SYN	/	1:100
C-CASP3	1:1000	/	HRP-conjugated IgG	1:5000	/
MITF	1:400	/	IgG H&L (Alexa Fluor® 488)	/	1:10000
mTOR	1:1000	/	IgG H&L (Alexa Fluor® 647)	/	1:10000

Table S3. Diluted concentration of antibodies for WB and IF staining.
